# Pot-Pollen Volatiles, Bioactivity, Synergism with Antibiotics, and Bibliometrics Overview, Including Direct Injection in Food Flavor

**DOI:** 10.3390/foods13233879

**Published:** 2024-11-30

**Authors:** Patricia Vit, Maria Araque, Bajaree Chuttong, Enrique Moreno, Ricardo R. Contreras, Qibi Wang, Zhengwei Wang, Emanuela Betta, Vassya Bankova

**Affiliations:** 1 Apitherapy and Bioactivity, Food Science Department, Faculty of Pharmacy and Bioanalysis, Universidad de Los Andes, Mérida 5101, Venezuela; 2 Laboratory of Molecular Microbiology, Department of Microbiology and Parasitology, Faculty of Pharmacy and Bioanalysis, Universidad de Los Andes, Mérida 5101, Venezuela; araquedpmc@gmail.com; 3 Meliponini and Apini Research Laboratory, Department of Entomology and Plant Pathology, Faculty of Agriculture, Chiang Mai University, Chiang Mai 50200, Thailand; 4 Smithsonian Tropical Research Institute, Calle Portobelo, Balboa, Ancon 0843-03092, Panama; morenoe@si.edu; 5 Department of Chemistry, Faculty of Science, Universidad de Los Andes, Mérida 5101, Venezuela; ricardo.r.contreras@gmail.com; 6 Yunnan Key Laboratory of Plant Reproductive Adaptation and Evolutionary Ecology, Institute of Biodiversity, Yunnan University, Kunming 650500, China; www5306@outlook.com; 7 CAS Key Laboratory of Tropical Forest Ecology, Xishuangbanna Tropical Botanical Garden, Chinese Academy of Sciences, Kunming 650033, China; wangzhengwei@xtbg.ac.cn; 8 Ricerca e Innovazione, Fondazione Edmund Mach, Via E. Mach 1, 38098 San Michele all’Adige, TN, Italy; emanuela.betta@fmach.it; 9 Institute of Organic Chemistry with Centre of Phytochemistry, Bulgarian Academy of Sciences, 1113 Sofia, Bulgaria; vassya.bankova@orgchm.bas.bg

**Keywords:** bibliometrics, biological activity, meliponini, metabolites, pot-pollen, stingless bee nest materials, synergism with antibiotics, volatile organic compounds

## Abstract

Stingless bees (Hymenoptera; Apidae; Meliponini), with a biodiversity of 605 species, harvest and transport corbicula pollen to the nest, like *Apis mellifera*, but process and store the pollen in cerumen pots instead of beeswax combs. Therefore, the meliponine pollen processed in the nest was named pot-pollen instead of bee bread. Pot-pollen has nutraceutical properties for bees and humans; it is a natural medicinal food supplement with applications in health, food science, and technology, and pharmaceutical developments are promising. Demonstrated synergism between *Tetragonisca angustula* pot-pollen ethanolic extracts, and antibiotics against extensively drug-resistant (XDR) bacteria revealed potential to combat antimicrobial resistance (AMR). Reviewed pot-pollen VOC richness was compared between Australian *Austroplebeia australis* (27), *Tetragonula carbonaria* (31), and *Tetragonula hogkingsi* (28), as well as the Venezuelan *Tetragonisca angustula* (95). Bioactivity and olfactory attributes of the most abundant VOCs were revisited. Bibliometric analyses with the Scopus database were planned for two unrelated topics in the literature for potential scientific advances. The top ten most prolific authors, institutions, countries, funding sponsors, and sources engaged to disseminate original research and reviews on pot-pollen (2014–2023) and direct injection food flavor (1976–2023) were ranked. Selected metrics and plots were visualized using the Bibliometrix-R package. A scholarly approach gained scientific insight into the interaction between an ancient fermented medicinal pot-pollen and a powerful bioanalytical technique for fermented products, which should attract interest from research teams for joint projects on direct injection in pot-pollen flavor, and proposals on stingless bee nest materials. Novel anti-antimicrobial-resistant agents and synergism with conventional antibiotics can fill the gap in the emerging potential to overcome antimicrobial resistance.

## 1. Introduction

**Figure 1:**
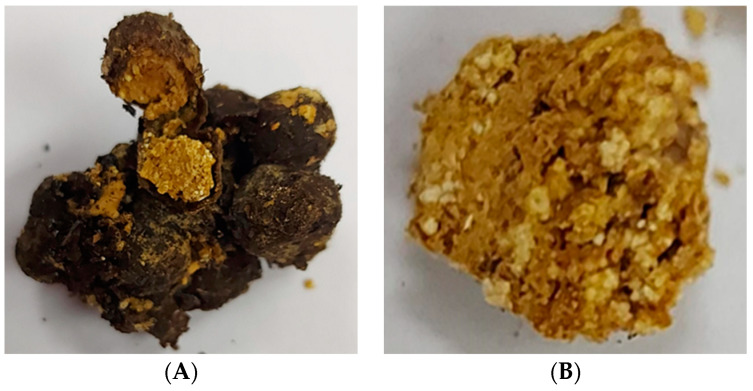
*Trigona corvina* pollen pots from Panama. (**A**) An assemblage of pollen pots harvested from a *Trigona corvina* nest with sliced cerumen of pollen pot to remove the content and (**B**) fermented pot-pollen mass removed from the cerumen pot. Different colors of pollen represent diverse botanical origins. Photos: ©E. Moreno.

A global biodiversity of 605 species of stingless bees, as described by Engel et al. (2023) [1], have ecological roles and beneficial uses as natural products. Stingless bees (Hymenoptera; Apidae; Meliponini) collect tropical floral pollen, mix it with nectar and salivary secretions, form pollen loads accommodated in the corbiculae, transport them to the nest, take fresh pollen into storage pots made of cerumen—an admixture of plant resins and stingless bee wax–and operculate the cerumen pot container for processing pot-pollen (Figure 1). Chemical, biochemical, and microbial transformations of floral pollen produce nutritional and sensory changes. Therefore, distinctive nutritional phytochemicals occur in this complex protein-rich nest material according to the botanical origin and metabolites derived from associated microbes with the stingless bee species. *Stingless bees process honey and pollen in cerumen pots* is the title of an e-book [2] published after *Pot-honey, A legacy of stingless bees* [3], and before *Pot-pollen in stingless bee melittology* [4].

Almost 1500 genera of tropical plants with diverse habits (herbs, trees, shrubs, vines, lianas, epiphytes), which have been selected for agricultural crops, the timber industry, fibers, medicinal sources, and ornamentals, offer resources to stingless bees; among them, the most visited families are Fabaceae, Asteraceae, Rubiaceae, Malvaceae, Lamiaceae, Euphorbiaceae, Arecaceae, Poaceae, Apocynaceae, and Melastomataceae [5]. Stingless bees’ more frequent interactions with available native plants are based on preferences of diverse origin evolving with natural history [6]. Pollen is known to cause allergies in humans via ocular or nasal mucosae penetration, but whether it is ingested fresh or dehydrated, alone or mixed with other nutraceuticals, or injected into pharmaceutical preparations has an antiallergenic action (A. Meléndez, personal communication [7]). Possibly, the bee processing of floral pollen may contribute to inactivating or removing the allergens (P. Vit observation). The use of pot-pollen by humans has nutritional benefits with functional properties, as shown by Rebelo et al. (2021) [8], and it also has antioxidant-based medicinal properties that have been recently reviewed [9,10,11,12].

The first published research on the chemical composition of stingless bee pollen in the Scopus database is of the last decade [13]. Although Fernandes-da-Silva and Serrão (2000) [14] compared the nutritive value and the apparent digestibility of bee-collected and bee-stored pollen of the Brazilian *Scaptotrigona postica* documented, they used a physiological method based on the development of the hypopharyngeal glands. Therefore, the physicochemical analyses of *Melipona mandacaia* pot-pollen by Barbara et al. (2015) [15] is the first document on this topic. Further interest was given to the protein content of Argentine *Geotrigona argentina*, *Melipona orbignyi*, and *Tetragonisca fiebrigi* [16], as well as proximate analyses of Venezuelan *Melipona* aff. *eburnea* and *Scaptotrigona* cf. *ochrotricha* [17] one decade later. Interest has also been given to the chapters in the *Pot-pollen in stingless bee melittology* book on *Scaptotrigona mexicana* [18]; Thai *Lepidotrigona flavibasis*, *Lepidotrigona terminata*, *Tetragonula laeviceps* complex, and *Tetragonula testaceitarsis* [19]; Brazilian *Melipona scutellaris* [20]; Venezuelan *Tetragonisca angustula* [21]; *Frieseomelitta* sp. aff. *varia*, *Melipona compressipes*, *Melipona eburnea*, *Melipona favosa*, *Melipona* sp. *fulva* group, *Melipona lateralis kangarumensis*, and *Melipona paraensis* [21]; and the most recent, *Tetragonula biroi* from the Philippines [22], Brazilian *Melipona interrupta* and *Melipona seminigra* [8], and *Melipona scutellaris* [23]. Protein-rich pot-pollen is a nutritional food for bee colonies, animals, and humans, and odors also attract pests like the small hive beetle *Aethina tumida* in *Trigona*, *Meliponula*, and *Dactylurina* stingless bee colonies [24]. Therefore, the study of pot-pollen volatility has growing interest in chemical ecology and sensory science.

Direct injection is a term unfamiliar to tropical food scientists. However, direct-injection mass spectrometric (DIMS) technologies are growing fast, combining mass and time resolution with high sensitivity and robustness [25]. These authors reviewed DIMS technologies used for rapid volatile organic compound (VOC) monitoring and quantitation, such as MS-e-noses, atmospheric pressure chemical ionization (APCI), proton-transfer-reaction mass spectrometry (PTR-MS), and selected ion-flow-tube mass spectrometry (SIFT-MS). APCI is an ionization method applied in environmental and flavor release. SIFT-MS and PTR-MS focus on precursor ion generation. SIFT-MS controls the ionization process, and PTR-MS improves sensitivity. Time-of-flight (ToF)-based equipment add further analytical power. Biasioli et al. (2011) [25] illustrated these key technologies for food quality control (MS-e-nose), flavor release (APCI), environmental sciences (PTR-MS), and health sciences (SIFT-MS). These multidisciplinary approaches would benefit from understanding potential pot-pollen applications. Indeed, direct injection and pot-pollen search queries retrieved no documents in the Scopus scientific database. The first interaction was during the International Symposium on Research and Innovation on Direct Injection Food Flavor Analytics (DIFFA), detecting *Tetragonisca angustula* pot-pollen VOCs from Venezuela [26].

The advantages of direct and non-destructive sampling by bioanalytical non-invasive PTR-MS benefit atmospheric chemistry, biological, environmental, and medical scientific research. An additional automated sampler for PTR-ToF-MS and tailored data analysis tools optimized applications to monitor bioprocesses such as enzymatic oxidation and alcoholic or lactic fermentation to screen large sets of diverse food origins or entire germoplasms, as well as to analyze variable VOCs in different formulations or variable processing factors [27].

The objective of this review was a multitask analysis of pot-pollen VOCs, their botanical, entomological, and microbial origin, their chemical diversity, olfactory attributes, nutritional and bioactive properties, synergism with antibiotics to slow antimicrobial resistance (AMR) for a novel anti-AMR activity, and pharmaceutic applications. The double bibliometrics approach for the stingless bee nest material and the powerful technique of direct injection in food flavor was performed to visualize their state of the art and potential interactive outcomes.

## 2. Botanical Diversity of Stingless Bee Pollen

Corbicula pollen loads and the pot-pollen of opened storage pots have visual heterogeneity because different botanical origins of floral pollen have different colors that also vary along the processing of pot-pollen in the nest. The botanical origin of pollen is identified by palynological analysis to determine the adistinctive morphologies of pollen grains with a light microscope [28]. The mature pollen powder of floral stamens has diverse colors and textures (powdery, sticky, waxy); under the microscope, colored oil droplets or the absence of oil can be observed. The pollen size varies from 10 to almost 300 µm, with a spheric, oblate, or prolate shape and surface ornaments, varying in the number of apertures and pores, being of furrow or colporate mixed types, and having monad or polyad arrays, which are all characteristic features used for botanical identifications by palynologists after comparing them with reference slide pollen collections and pollen atlases [29,30,31]. The environmental availability of pollen and stingless bees’ foraging preferences determine the palynological spectra, chemical composition, and biological activity of pot-pollen besides the microbial transformations in the nest. Behavior inside the nest may also add variations more relevant for honey contamination with polleniferous plants than pot-pollen itself contaminated with nectariferous plants, possibly present because pollen needs a small quantity of liquid nectar to amalgamate transportable pollen loads in the corbiculae (P. Vit, personal observation).

The great biodiversity of plants produce pollen with variable proximate macronutrients (ash, carbohydrates, fat, moisture, proteins), secondary metabolites (aliphatic organic acids, aldehydes, esters, fatty acids, flavonoids, ketones, oxides, polyols, polyphenols, terpenes, phytosterols, vitamins), minerals (nutritional K, Na, Fe, Mg, Mn, Zn, and contaminants Cd, Hg, Pb), and biological activity (antibacterial, anticancer, antidiabetic, anti-inflammatory, antioxidant). Pollen collected from plants has a microbial reservoir (bacteria, fungus, yeasts) selected somehow by stingless bees. This is known as microbial associations with stingless bees because a microbial spectrum has been isolated in diverse nest materials, including pot-pollen [32,33,34,35].

**Figure 2:**
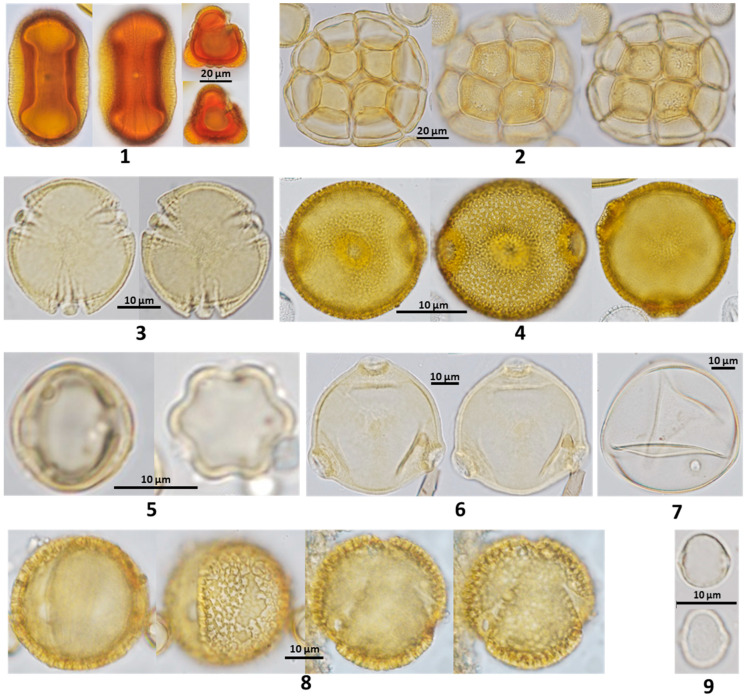
Equatorial and polar views of Eudicotyledoneae pollen grains. Pollen taxa of Neotropical plants is used by stingless bees. (**1**) Acanthaceae: *Asystasia gangetica*, (**2**) Fabaceae-Caesalpinioideae: *Acacia hayesii*, (**3**) Euphorbiaceae: *Alchornea latifolia*, (**4**) Malvaceae-Bombacoideae: *Quararibea asterolepis*, (**5**) Melastomataceae: *Miconia* sp., (**6**) Onagraceae: *Ludwigia* sp., (**7**) Poaceae: *Zea mays*, (**8**) Rubiaceae: *Posoqueria latifolia*, and (**9**) Urticaceae: *Cecropia* sp. 100× (photos not to scale). Photos: ©E. Moreno. After [38].

The exine is the outer resistant sporopollenin surface of a pollen grain, and its morphology is studied in palynology to identify the botanical origin (Figure 2). This structure contains nutritious material rich in enzymes needed for pollen germination. Bioactive molecules introduced in pollen capsules extend the antibody action in oral vaccinations [36]. A review on corbiculae *Apis mellifera* bee pollen and bee bread shows the potential of these natural products for human health [37].

Compared to other stingless bee products of the nest, the pollen-based materials are of visible pollen botanical origin, with minor contaminants that may occur in a busy trafficking bee nest. That of the bee bread for *Apis mellifera* or pot-pollen for Meliponini is the corbicula pollen being transformed for the nutritional functions of the bee colony. Other materials have pollen residues related to the nectar origin for honey, debated plant resin origin, or more complex interpretations for royal jelly, beeswax, and derived materials like propolis and stingless bee cerumen. And yet, the pollen was foraged by particular stingless bee species from the available environmental pollen and transformed by distinctive microbial associations with diverse bee species, involving floral pollen, corbicular pollen foraged and transported from floral anthers to the nest, and pot-pollen processed in the stingless bee nest.

In the first bibliometric review on stingless bee pollen, about half of the retrieved documents were on palynology [13]. Indeed, 47% of the documents were of the agricultural and biological sciences Scopus subject area with further research on biochemistry, genetics, and molecular biology (13.5%), environmental science (10.5%), medicine (4.3%), engineering (3.9%), and chemistry (3.3%), where chemical composition and biological activities were published including antibacterial, antioxidant, anticancer, antidiabetic, anti-inflammatory, and antinociceptive actions.

## 3. Chemical Diversity of Volatile Metabolites in Stingless Bee Pollen

### 3.1. Chemical Classes of Pot-Pollen Metabolites

The bioactive metabolites of stingless bee pollen comprise phytochemicals generally extracted with organic solvents (ethanol, ethyl acetate, methanol) and more recently with environmentally friendly headspace solid-phase microextractions, with a positive role for the direct mass spectrometric analytical option for volatilomics research. Silva et al. (2006) [39] analyzed non-volatile flavonoids and detected naringenin, isorhamnetin, D-mannitol, β-sitosterol, tricetin, selagin, and 8-methoxiherbacetin in *Melipona subnitida* pot-pollen from Brazil by high-performance liquid chromatography, HPLC. Omar et al. (2018) [40] compared derivatized pot-pollen ethanolic extracts of Malaysian *Geniotrigona thoracica*, *Heterotrigona itama*, and *Tetrigona apicalis* studied by GC-MS and found their main polyol sugar alcohol was mannitol (33.05–54.34%), with minor quantities of hydrocarbons such as propanoic acid and hexadecenoic acid (1.28–4.4%), as well as the polyunsaturated fatty acids linoleic acid and α-linolenic acid (0.07–1.11%). Belina-Aldemita et al. (2019) [22] compared the contents of total phenolics at 7.95–24.75 mg GAE/g (GAE, gallic acid equivalents), total flavonoids at 16.13–35.04 mg QE/g (QE, quercetin equivalents), total anthocyanins at 0.19–0.74 mg CGE/g (CGE, cyanidin 3-O-glucoside equivalents), and the flavonoids rutin at 0.47–1.55 mg/g and quercetin glucoside at 1.43–3.39 mg/g of *Tetragonula biroi* pot-pollen from the Philippines. Major volatile organic compounds (VOCs) of Australian stingless bee pot-pollen were assessed by HS-SPME-GC-EI-MS, and the results varied with the species [41]. *Austroplebeia australis* had no acetic acid, caryophyllene, (+)-ledene, epiglobulol, labd-14-ene, a sesquiterpene, or a hydrocarbon. *Tetragonula carbonaria* and *Tetragonula hogkungsi* had no 4-ketoisophorone, cis-geraniol, and α-citral. Additionally, *Tetragonula hogkingsi* lacked nonanal, cis-α-bisabolene, and a sesquiterpene. Major VOCs acetic acid, 2,3-butanediol, β-phellandrene, 2-methyl-1-propanol, propylene glycol, furfural, ethanol, and ethyl acetate of Venezuelan *Tetragonisca angustula* pot-pollen were compared by headspace solid-phase microextraction–gas chromatography–mass spectrometry (HS-SPME/GC-MS) with the cerumen pot [26].

Ten chemical classes of pot-pollen VOCs are presented in Table 1, grouped into acids, alcohols, aldehydes, esters, ketones, monoterpenes, oxides, sesquiterpenes, and others, including polyols.

Some metabolites were found in Australian and Venezuelan pot-pollen, for example, hexanal, caryophyllene oxide, and α-copaene. However, the majority are characteristic of each country; their presence or absence could be related to the vegetation. After comparing the three Australian stingless bee species, it is evidenced that the previously reported lack of acetic acid in *Austroplebeia australis* is related to the associated microbiota, clearly showing that acetic acid bacteria (AAB) are associated with *Tetragonula carbonaria* and *Tetragonula hockingsi* but not to *Austroplebeia australis* [41].

**Figure 3:**
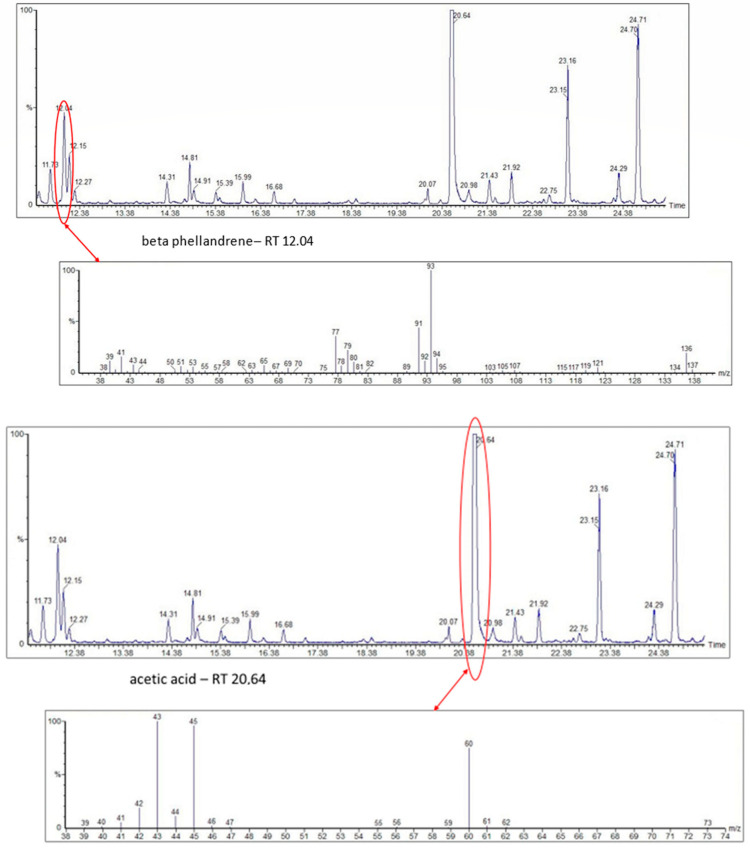

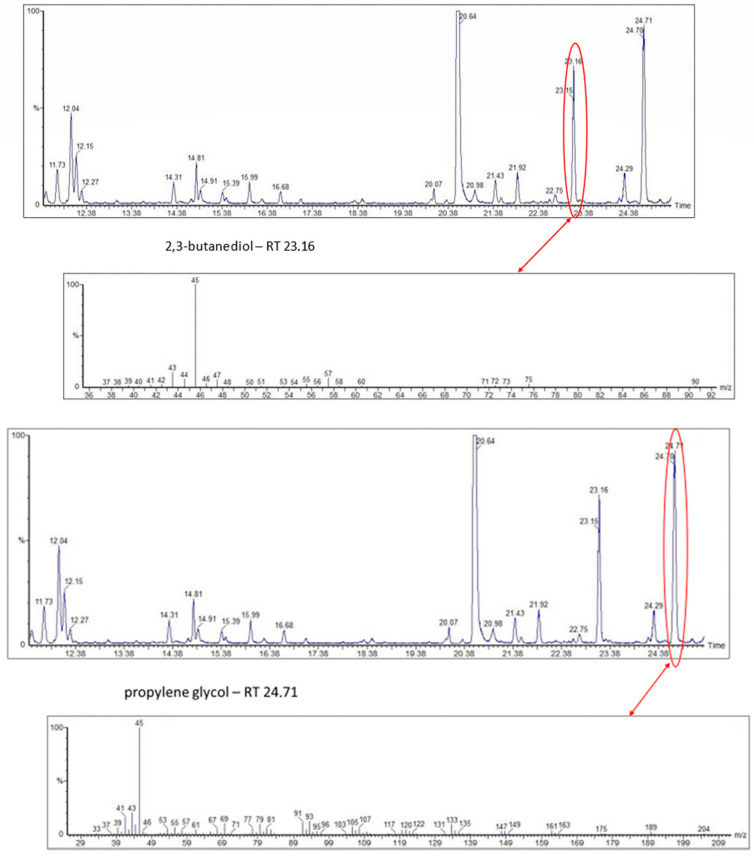
SH-SPM/GC-MS spectra to visualize acetic acid, 2–3, butanediol, β-phellandrene, and propylene glycol of *Tetragonisca angustula* pot-pollen from Mérida, Venezuela. Graphic design: ©E. Betta.

In Figure 3 are illustrated the GC chromatograms and MS spectra of the four most abundant VOCs of *Tetragonisca angustula* pot-pollen, the microbial acetic acid that confers the distinctive organoleptic smell and sour taste, the suspected microbial origin of polyol 2–3, butanediol, the plant origin monoterpene β-phellandrene, and propylene glycol, which is the second suspected polyol of microbial origin.

### 3.2. Odorants of Pot-Pollen 

Volatile organic compounds (VOCs) are signaling molecules in insect communication, microbial processes, and survival, and they are perceived by humans in contact with ants, stink bugs, and bees, to mention familiar examples. The biodiversity of 605 stingless bee species [1] has distinctive sensory attributes, such as the blue cheese smell of the *Scaptotrigona vitorum* nest in Ecuador, but the chemical nature and the ecological role of that VOC have not been elucidated, as well as the suspected yeast origin [42].

Olfactory neuron receptors perceive odors. The bouquet is a complex relationship of multivariate factors. For example, ethanol contents determine the perception of aroma attributes and the detection of VOCs in Malbec wine, causing herbaceous smell perception at about 15% and a fruity perception at lower ethanol concentrations [43]. Tasting ethanol produces a burning mouthfeel sensation by the trigeminal nerve, and although familiar, it does not have a smell-like descriptor, with ethanol being the descriptor itself. Indeed, detecting an ethanol smell is an indicator of fermentation, such as that caused during the post-harvest of *Scaptotrigona mexicana* honey associated with increased healing power [44]. In Table 2 are described the olfactory attributes of some pot-pollen VOCs from Table 1 and the food, microbial, or plant origins of these metabolites.

## 4. Nutritional Composition and Biological Activities of Pot-Pollen

Proximal analyses provide core chemical components used in nutritional tables, expanded with contents of minerals and vitamins. Pollen is the richest source of proteins, free amino acids, lipids, free fatty acids, minerals, and vitamins for bees [45]. The classic anti-inflammatory, antimicrobial, antimutagenic, and antioxidant biological activities reviewed for corbicula *Apis mellifera* bee pollen, as described by Pascoal et al. (2014) [46], have been reviewed here for stingless bee pot-pollen, a potential food supplement for its nutraceutical properties.

### 4.1. Nutritional Facts of Pot-Pollen, Still Named Stingless Bee Bread, Which It Is Not

Let us consider that in the Standard for honey [47], honey is only produced by *Apis mellifera*, so bee bread is produced only by *Apis mellifera*. Storage structures of *Apis mellifera* are beeswax combs, built with a different material and shape than the cerumen pots used by diverse species of stingless bees [3,48]. Both bee bread and pot-pollen are the protein source for the honeybee and stingless bee colony, respectively. Proximal analyses of pot-pollen were revised by Vit et al. (2018) [49], considering the data of 18 stingless bee species from Brazil (*Melipona interrupta*, *Melipona mandacaia*, *Melipona scutellaris*, *Melipona seminigra*), Mexico (*Scaptotrigona mexicana*), Thailand (*Lepidotrigona flavibasis*, *Lepidotrigona terminata*, *Tetragonula laeviceps*, *Tetragonula testaceitarsis*), and Venezuela (*Frieseomelitta* sp. aff. *varia*, *Melipona eburnea*, *Melipona* sp. aff. *eburnea*, *Melipona favosa*, *Melipona* sp. *fulva* group, *Melipona lateralis kangarumensis*, *Melipona paraensis*, *Scaptotrigona* sp. cf. *ochrotricha*, *Tetragonisca angustula*); they are briefly summarized here as tentative pot-pollen standards considering contents of ash < 5%, carbohydrates 15–55%, lipids > 1.5%, moisture < 30%, and proteins > 15%. However, variations in this natural product demand careful attention related to the botanical origin of foraged resources, habitat, climate, soil type, stingless beekeeper handling, dehydration to extend shelf-life, storage conditions of temperature and relative humidity, and other treatments for marketing.

Pot-pollen is known to be generally a moist material, sometimes having multilayered pollen of different colors within a single pot and others transformed into a dark paste. The ash content comprised between 1.8% Thai *Lepidotrigona terminata* and 4.9% Brazilian *Melipona mandacaia*. Potassium, calcium, and magnesium were the most abundant minerals, with traces of zinc, manganese, iron, and copper in Brazilian Melipona subnitida pot-pollen [50]. The moisture varied from 16.1% Thai *Tetragonula laeviceps* to 48.5% Venezuelan *Melipona* sp. aff. *eburnea* [49]. Carbohydrate content in South East Asia varied from 43.1% Thai *Tetragonula testaceitarsis* to 59.9% Philippine *Tetragonula biroi*, as shown by Mohammad et al. (2021) [51], but from 24.5% Brazilian *Melipona scutellaris* to 58.7 Thai *Tetragonula laeviceps*, as shown by Vit et al. (2018) [49]. Mannitol content was 20.8% to 31.0% in Brazilian Jandaira *Melipona subnitida* [50]. Protein content ranged from 14.3% Thai *Lepidotrigona terminata* to 24.7% Venezuelan *Frieseomelitta* sp. aff. *varia* and had 1.83 g/100 g total free amino acids of Philippine *Tetragonula biroi* [22]. Proline and serine were the predominant amino acids (56% of total free amino acids) and tryptophan was absent in *Melipona subnitida* pot-pollen from Brazil [50]. Lipid contents varied between 0.89% Mexican *Scaptotrigona mexicana* and 7.4% Thai *Tetragonula laeviceps* [49]. According to Szczesna (2006) [52], linoleic (omega-6), linolenic (omega-3), and palmitic acids were the most abundant fatty acids in honeybee pollen.

The total flavonoids and polyphenols are quantified with spectrophotometric methods, and liquid chromatography (LC) is mostly used for identifications using diverse detectors such as ultraviolet light (UV) and mass spectrometry (MS). These secondary metabolites of plants participate in biological activities. The flavonoids of pot-pollen are produced by diverse species of stingless bees (*Frieseomelitta* sp. aff. *varia*, *Melipona compressipes*, *Melipona eburnea*, *Melipona favosa*, *Melipona* sp. *fulva*, *Melipona lateralis kangarumensis*, and *Melipona paraensis*) were studied in Southern Venezuela [49]. Phenolic compounds were highly correlated with the antioxidant activity of Philippine *Tetragonula biroi* pot-pollen [53] and with the anti-atherogenic effect of Malaysian *Heterotrigona itama* pot-pollen in high-fat diet-induced obese rats [54].

### 4.2. Biological Activities of Most Abundant VOCs in Pot-Pollen

The most abundant Venezuelan *Tetragonisca angustula* pot-pollen volatile metabolites shown in Table 1 are presented in Table 3 with their chemical structures, relative abundance, microbial or plant origin, and biological activity.

Selected Australian volatile metabolites of *Austroplebeia australis*, *Tetragonula carbonaria*, and *Tetragonula hockingsi* pot-pollen of Table 1 are presented in Table 4 with their chemical structures, microbial or plant origin, and biological activity.

### 4.3. Promising Stingless Bee Resources with Biological Activities

Pot-pollen has antibacterial and antioxidant properties. A screening of ethanolic extracts of Venezuelan *Frieseomelitta* aff. *varia*, *Melipona compressipes*, *Melipona eburnea*, *Melipona favosa*, *Melipona* sp. group *fulva*, *Melipona lateralis kangarumensis*, *Melipona paraensis*, and *Tetragonisca angustula* pot-pollen produced inhibition zones from 8.5 to 14.0 cm against Gram-positive bacteria *Bacillus subtilis* and *Staphylococcus aureus* and from 10.5 to 13.0 cm in Gram-negative bacteria *Enterobacter cloacae*, *Escherichia coli*, and *Pseudomonas aeruginosa* [84] in the Brazilian Amazon [85]. The antioxidant activity is measured in vitro for the organic extracts of the studied materials, which is pot-pollen in this review, using a battery of methods responding to diverse mechanisms of action and using reagents like the ABTS^•+^ 2,2′-azinobis-3-ethylbenzotiazoline-6-sulfonic acid and the DPPH^•^ 2,2-diphenyl-1-picrylhydrazyl radical. The ferric-reducing antioxidant power, known as FRAP, the oxygen radical absorbance capacity, ORAC, the Trolox equivalent antioxidant capacity, TEAC, and the hydroxyl radical inhibition are also used. The antioxidant activity of *Melipona subnitida* pot-pollen extracts varied according to the organic solvent; thus, the active metabolites were extracted more efficiently with ethyl acetate than ethanol and hexane [39]. The hydroxyl radical inhibition (% inhibition/100 g pot-pollen), antioxidant activity AOA (mM equivalent uric acid/100 g), and the TEAC (μmoles Trolox equivalents/100 g pot-pollen) methods were used to measure the antioxidant activity in ethanolic extracts of *Austroplebeia australis* (58.0, 1.01, 248.8), *Tetragonula carbonaria* (49.3, 1.04, 203.0), and *Tetragonula hockingsi* (74.8, 1.06, 430.7) pot-pollen from Australia, respectively, and the activity was highest for *Tetragonula hockingsi* using the three methods [86]. The antioxidant activity of hydroethanolic extracts of the Brazilian Tubí *Scaptotrigona* aff. *postica* pot-pollen was measured with ABTS IC_50_ 87.29 µg/mL, DPPH IC_50_ 273.08 µg/mL, and a ferric-reducing antioxidant power, FRAP, of 0.71 mmol Fe^2+^/g [87]; for the Brazilian *Melipona seminigra*, FRAP varied from 2.80 to 8.90 μmol Trolox equivalents and ORAC from 224.90 to 1117.00 μmol Trolox equivalents [8].

Antimicrobial activity and active microbial metabolites seem like a paradox, but they are not. The beneficial microbes produce metabolites to combat microbial pathogens evaluated in antimicrobial activity. The microbiota of pot-honey and pot-pollen of *Scaptotrigona jujuyensis* produced hydrolytic enzymes such as protease, amylase, xylanase, cellulase, and lipase. An isolated *Bacillus* sp. produced an extracellular exopolysaccharide (EPS) similar to levan, with antimicrobial activity and emulsifying hydrogel formation capacity with omega-3 polyunsaturated fatty acids (PUFAs) from ray liver and chia oils [88].

Extracts of pot-pollen have demonstrated anti-inflammatory and anticancer action. The oral administration of hydroethanolic extract prepared with 250 mg/kg Tubí *Scaptotrigona* aff. *postica* pollen from Brazil needed 5 h to reduce 100% carrageenan-induced and dextran-induced paw edema in mice [87]. Indonesian *Tetrigona apicalis, Tetragonula incisa, Tetragonula fuscibasis*, and *Tetragonula fuscobalteata* pot-pollen ethyl acetate extracts were more cytotoxic against lung undifferentiated cancer ChaGo-I and ductal carcinoma BT474 cell lines than *n*-hexane and methanolic extracts [89]. A synergistic antiproliferative effect was observed between Malaysian *Lepidotrigona terminata* pollen extracts and the anticancer drug cisplatin on the MCF-7 breast cancer cell line compared to cisplatin alone based on the MTT assay data assessed; thus, pot-pollen is a potential chemopreventive agent, reducing about 50% of the therapeutic cisplatin dose [90]. 

Metabolic syndromes also received the attention of pot-pollen scientists. Othman et al. (2020) [54] studied the effect of dietary Malaysian *Heterotrigona itama* pot-pollen in a model of high-fat diet-induced obese rats. This pot-pollen reduced the negative health indicators of the obesity index, total cholesterol, low-density lipoprotein, fatty acid synthase activity, the atherogenic index, oxidized-LDL, and malondialdehyde; and significantly influenced positive indicators in the aorta such as an increased antioxidant enzyme activities of superoxide dismutase and glutathione peroxidase, smaller adipocyte sizes, and an absence of atherosclerotic plaque. Rebelo et al. (2022) [91] observed that Brazilian *Melipona seminigra* pot-pollen supplementation decreased fasting blood glucose and increased glucose-stimulated insulin secretion in high-fructose diet-induced obesity in C57BL/6J mice. Additionally, dietary pot-pollen modulated gut microbiota in correlation to the observed decrease in fasting blood glucose in mice.

Antinociceptive properties were demonstrated for a hydroethanolic extract (500 mg/kg) of Tiúba *Melipona fasciculata* pot-pollen reducing the biting/licking time as an indicator of pain detection in mice after the application of acetic acid and formalin tests, similar to indomethacin analgesic control inhibiting histamine release and decreasing the synthesis of prostaglandins [92]. Pot-honey polyphenols and fatty acid phytochemicals were the potentially active metabolites according to their in silico study.

A tropical infectious disease has promising therapy for the leishmanicidal activities of Brazilian Jupará *Melipona compressipes manaosensis* pot-pollen from Maues, Amazonas, and it was assessed after testing crude maceration extracts using hexane, followed by ethanol, then methanol for 24 h, against *Leishmania* (Viannia) *guyanensis* (M 4147) and *Leishmania naiffi* (M 5533). Larvicidal activity was evaluated on *C ulex quinquefasciatus* and the LC_50_ after 48 h was about 200 μg/mL [85]. The effect of the reference hepatoprotective drug silymari has been compared to the action of bee bread [93] and could be possibly investigated for pot-pollen.

### 4.4. Pot-Pollen Is Emerging as a Potential Synergistic Agent Boosting the Efficacy of Conventional Antibiotics to Overcome Antimicrobial Resistance

Antimicrobial resistance (AMR) is a significant growing concern threatening worldwide public health [94,95]. Therefore, the World Health Organization (WHO) has highlighted the imperative need for novel approaches to address this soaring threat. Among these, natural products such as pot-pollen and its derivatives have emerged as a promising source of active compounds as insect medicinal alternatives—particularly meliponitherapy—with anti-AMR activity. Only two documents on pot-pollen were retrieved from the Scopus database in 2024, and there were no publications in clinical journals [96].

The World Health Organization (WHO) updated the list of the twelve bacteria that should be considered a priority for the research and development of new antibiotics due to their impact on public health and the limited treatment options available [95]. The WHO list is divided into three categories based on the urgency with which novel therapeutic alternatives are needed, namely critical, high, and medium priority categories. The critical priority group encompasses multidrug-resistant (MDR) and extensively drug-resistant (XDR) bacteria, which represent a significant threat in hospital settings, nursing homes, and long-term care units, as well as among patients requiring device-based care, such as ventilators and intravenous catheters. These are bacteria with documented resistance to third-generation cephalosporins and carbapenems, which can lead to severe infections, including sepsis and pneumonia, with high mortality rates [94,95]. The emergence of antimicrobial resistance (AMR) has highlighted the need for new therapeutic approaches to address this growing threat [94], among which natural products such as pollen and its derivatives have emerged as a promising source of active compounds with antimicrobial activity.

Recent reports indicate for the first time that an ethanolic extract of pot-pollen from Venezuelan *Tetragonisca angustula* has the potential to reduce the minimum inhibitory and minimum bactericidal concentrations of amikacin and meropenem against six extensively drug-resistant Gram-negative bacteria [97]. This synergistic activity is attributed to the multiple bioactive compounds present in pot-pollen, including antimicrobial peptides, fatty acids, flavonoids, and microbial-derived compounds to be deciphered with characterizations of microbiomes associated with stingless bees. For example, microbiota of *Scaptotrigona jujuyensis* pot-pollen from Argentina produced enzyme-rich material containing proteases, amylases, xylanases, cellulases, and lipases [88]. All those compounds, when associated with antibiotics, broaden the spectrum of action and intensify their biological mechanisms [13,97]. The anti-AMR efficacy of pot-pollen produced by one of the 605 global stingless bee species, elucidated by Engel et al. (2023) [1], combined with two profusely and clinically used antibiotics could be compared with research conducted for *T. angustula* pot-pollen in other countries, as well as involving a greater number of pantropical stingless bee species, habitats, other materials of the nest, as well as a wider range of antibiotics.

It is possible to develop therapeutic protocols to fight antimicrobial resistance based on safe clinical studies and immune-boosting strategies in communities where pot-pollen is regularly produced and consumed [13,98]. Moreover, the spectrum of resistant bacteria under investigation could be extended to include those rated as a critical priority due to their substantial impact on public health. Additionally, it has been reported that compounds present in bee products not only inhibit the growth of pathogenic microorganisms but may also positively influence the host microbiota, acting in a prebiotic manner [99].

A further avenue of research could involve the utilization of pot-pollen extracts in pharmacological procedures with the objective of optimizing the clinical efficacy of antibiotics, reducing drug doses, and minimizing side effects. Bee pollen is a functional food and a scientifically validated nutritional candidate with experimental and preclinical evidence projecting towards relevant clinical insights to readjust homeostasis [99]. Clinical trials designed for allergies, prostatitis, cancer, and skin problems [100] could support clinical trial design, aiming to reveal a synergism of pot-pollen with antibiotics against a spectrum of antibiotic-resistant bacteria, including the twelve prioritized multidrug-resistant (MDR) and extensively drug-resistant (XDR) bacteria magnifying risks to public health [95]. Similarly, studies aimed at defining the microbiota associated with the pot-pollen substrate would facilitate an understanding of the biological and genetic interrelationships occurring in this microhabitat, eventually defining it as “active pot-pollen” for its richness in biomolecules of microbial origin [97].

Investigating the molecular mechanisms of antimicrobial resistance, the impact on clinical practices, and the socio-economic factors influencing the spread of resistant strains, besides the interest in innovative strategies for surveillance and novel therapeutic approaches were the aims of the Special Issue: Challenges and Opportunities in Antibiotic Resistance of the journal *Medical Research Archives* (2024). It was a wise call for a multidisciplinary dialog on actions to overcome antibiotic resistance with advancing treatment and prevention strategies. Stingless bee products, particularly pot-pollen, have a biodiversity of emerging potential both in preventive and therapeutic settings to mitigate the incidence of antimicrobial resistance. The lack of research in this topic was evidenced by Vit et al. (2024) [13] and Vit et al. (2024) [96] after no documents were retrieved in the Scopus search of pot-pollen AND synergism AND antibiotic within the field title, abstract, and keywords; they demanded an editorial effort with a book proposal to screen the synergism of global pot-pollen with conventional antibiotics to fill the gap in the emerging threat to overcome antimicrobial resistance in a submitted book proposal (*Novel anti-antimicrobial-resistant agents*) to Springer in 2024.

The integration of *T. angustula* pot-pollen ethanolic extracts into existing antibiotic regimens represents a promising strategy that may facilitate the treatment of XDR bacterial infections. Its capacity to reduce antibiotic doses, combat AMR, and function as a broad-spectrum agent has the potential to transform current approaches to managing bacterial infections and address the global AMR crisis [97]. To fully capitalize on these advantages and translate them into effective clinical applications, further research, preclinical, and clinical trials are essential.

Although studies in this field are scarce, further research will not only expand knowledge about these products but may also yield new strategies in alternative medicine and meliponitherapy. A comprehensive understanding of the composition, mechanisms of action, and interactions with the microbiota are crucial for fully harnessing the potential of stingless bee nest (SBN) materials in human and animal health.

## 5. Bibliometrics on Pot-Pollen and Direct Injection in Food Flavor Research

The classic metrics of the best ranked authors, institutions, countries, sources, and funding sponsors to create visualizations of Bibliometrix plots was the selected method for this objective. The Scopus database is the most complete and largest scientific database, according to Falagas et al. (2008) [101], and it was available to the authors. The ten most productive authors, institutions, countries, sources, funding sponsors, and subject areas were tabulated in descending order of the number of documents and alphabetical order for those sharing the same number of documents. For the top ten sources, the h index, quartile, and impact factors of journals were consulted online with Resurchify.

The Bibliometrix software in R Version 4.2.1 and the Biblioshinny (Stable Version, https://www.bibliometrix.org/home/index.php/download) builded in the most recent version of R (R 4.4.2, https://www.r-project.org/) permit multivariate analyses and optimized visualizations [102]. They were applied to the dataset exported as a comma-separated-value (csv) Excel file from the Scopus database. Two bibliometric reviews on stingless bee nest materials and the analytical technique were performed, and then pot-pollen and direct injection in food flavor metrics were compared.

### 5.1. Bibliometrics on Pot-Pollen Research

The bibliometric search and top ten rankings were performed using the Scopus database in the “TITLE-ABS-KEY” field on 15 November 2023. The operators AND and AND NOT of all documents were used in the following query string:

TITLE-ABS-KEY (pot-pollen AND NOT fossil).

After the Scopus rankings, Bibliometrix-R tool plots were selected and visualized from the csv file using authors’ keywords, keywords Plus, co-authors’ collaborative networks, the country collaborative map, the conceptual structure for the highest contributions, and the most globally cited documents.

The dataset retrieved from the Scopus database on pot-pollen research consisted of 40 documents. The main information of bibliometric descriptors is presented in Table 5, including publications from the first retrieved document, from 2014 to 2023.

#### 5.1.1. Most Productive Authors in Pot-Pollen Research

The top ten authors in Table 6 are from Venezuela (2), Brazil (2), Indonesia (1), the Philippines (1), Austria (2), and Malaysia (2), with six to two documents in the period of 2014–2023.

#### 5.1.2. Geographical Distribution of Productive Institutions and Countries in Pot-Pollen Research

In Table 7, the top ten institution from Venezuela (1), Australia (1), Brazil (4), Austria (2), Indonesia (1), Malaysia (1), and the Philippines (1) were ranked from three to seven documents published since 2014.

The ten most productive countries on stingless bee pollen and pot-pollen research are ranked in Table 8. The countries that conducted pot-pollen research produced 3 to 15 documents each. The top five countries were from South America, Oceania, and Asia, as well as Brazil, Australia, Venezuela, Indonesia, and Malaysia. Ecuador, the Philippines, Argentina, Austria from Europe, and Mexico from North America were included in the next five countries.

#### 5.1.3. Most Frequently Used Sources for the Dissemination of Research in Pot-Pollen Research

Table 9 shows the top ten sources used by authors to publish their research on pot-pollen from 2013 to 2023. Each of these journals hosted between sixteen and one document each. The top five most productive sources were the book *Pot Pollen in Stingless Bee Melittology*, the *Iop Conference Series Materials Science and Engineering*, and the *journals Journal of Apicultural Research*, *Livestock Research for Rural Development*, and *Neotropical Entomology*. The journals’ h index varied between 138 and 20; 2/8 journals are in Quartile 1. The maximum impact score was 4.56 for the journal *Drying Technology*, ranked in the ninth position of the dataset.

#### 5.1.4. Main Funding Sources in Pot-Pollen Research

The funding sponsors supporting pot-pollen research are ranked in Table 10 according to the number of publications. Four of the top ten funding agencies that sponsored pot-pollen research were from Brazil, two from Malaysia, two from Austria, and one from both Denmark and Argentina.

#### 5.1.5. Main Subject Areas of Research in Pot-Pollen Research

Table 11 shows that the top ten Scopus subject areas of pot-pollen research since 2014 are mostly in agricultural and biological sciences for 32.7% of the documents. The following Scopus subject areas of interest were engineering (19.8%), biochemistry, genetics, and molecular biology (18.8%), environmental science (16.8%), materials science (4.0%), and chemistry (2.0%).

#### 5.1.6. Authors’ Keywords and Keywords Plus: Most Relevant Words and Dendrogram in Pot-Pollen Research

**Figure 4:**
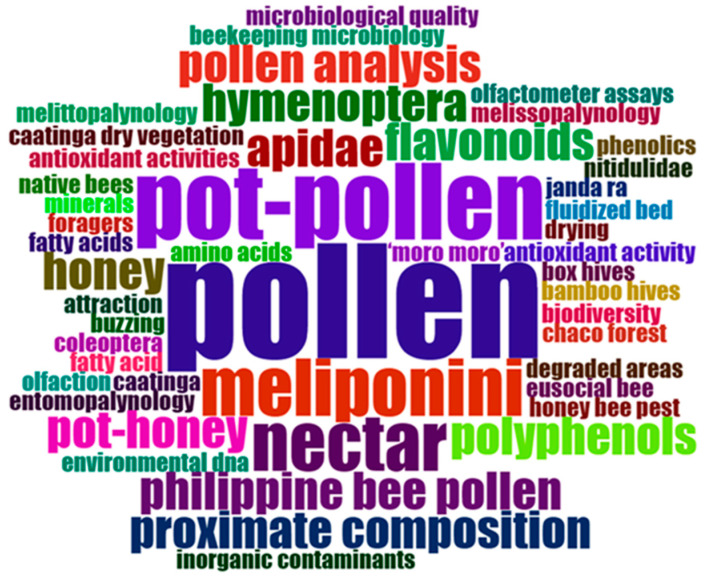
Word cloud by author keywords in the Scopus dataset of pot-pollen since 2014.

In Figure 4, a word cloud plot was based on 50 author keywords in documents of the pot-pollen dataset since 2014, with a frequency order from 1 to 11 occurrences. The frequencies of author keywords correspond to different colors, position, and size. The largest and most central keywords are the most frequent and were visualized in this plot for pollen (11), pot-pollen (6), meliponini (4), and nectar (3).

**Figure 5:**
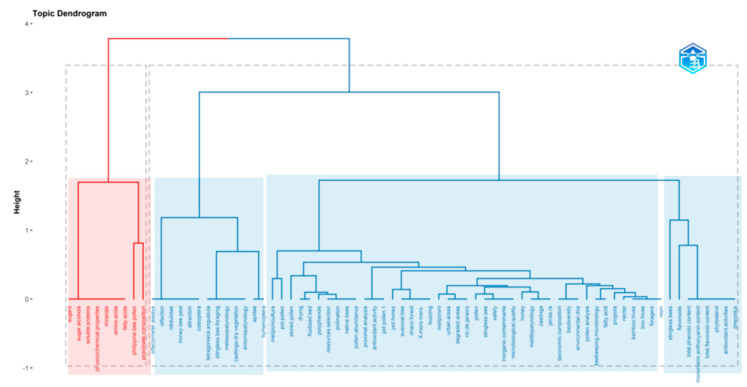
Topic dendrogram by HCA of keywords Plus in pot-pollen publications since 2014. The suggested topics for the red cluster are nutritional factors including sugars, sugar alcohols, soluble proteins, physicochemical properties, minerals, amino acids, and fatty acids, mostly primary metabolites. For the blue cluster two branches on biodiversity, palynology, pollination, and secondary metabolites, as well as countries, are visualized.

In Figure 5, a dendrogram was built using a hierarchical cluster analysis (HCA) of keywords Plus. Note that keywords’ style of Bibliometrix is lowercase and italics are not used. The nutritional information was grouped in the red cluster comprising the following keywords: sugars, sugar alcohols, soluble.proteins, physicochemical.properties, minerals, amino.acids, fatty.acids, philippine.bee.pollen, and proximate analysis. The larger blue cluster was divided into three branches and covered topics on biodiversity, palynology, pollination, secondary metabolites, and countries. 1.1 olfactometer assays, olfaction, nitidulidae, honey.bee.pest, attraction, and coleoptera; 1.2 tetragonisca.angustula, stingless.bee.foraging, melissopalynology, caatinga.dry.vegetation, entomopalynology, and 1.3 apidae, hymenoptera; 2. this is the largest cluster with pot-pollen and pot-honey; 2.1 meliponiculture, drying, fluidized.bed, polyphenols, resources selection, pollination, native.bees, native.bees, and pollen.abundance; 2.2 proximal.analysis, pot-pollen 1, antioxidant.activity, pot-honey, eusocial.bees, chaco.forest, moro-moro, buzzling, meliponini, urban.areas, degraded.areas, rio.de.janeiro, pollen, stingless.bees, safety, inorganic.contamination, microbiological.quality, honey, melittopalynology, caatinga, and jandaira; 2.3 taxonomic.composition, biodiversity, environmental dna, pollen analysis, beekeeping.microbiology, fatty acid, propolis, nectar, bamboo.hives, box.hives, foragers, and resin; 3. pot.pollen and pot.honey; 3. stingless.bees, flavonoids, total.phenolic.contents, monomeric anthocyanin.content, total.flavonoid.content, phytosterol, antioxidant.activities, and phenolics.

The first comment is that scientists are not experts in keyword selection, including the authors of this review, as well as journal editors. A modern instruction for authors suggests not including words of the title as keywords, which is rather absurd when considering a bibliometrics search using titles and keywords, or titles, abstracts, and keywords, as in our query string. If an author has pot-pollen in the title, then they are forced to use another expression in the keywords instead of reinforcing pot-pollen in this case. For this reason, stored.pollen is used as a keyword instead of pot.pollen. In this dataset, there are pot-pollen and pot-pollen.1, but it is impossible to know what the second expression is. A second comment is the use of plurals; if a rule advised it is enough to have stingless bees, because stingless bees is considered another keyword, which it is not, surely editors and authors would follow that. Some conceptual words like melittopalynology and melissopalynology, having the same meaning, must be unified into one of them as recommended and the other discarded; perhaps palynology would be a better choice valid for analysis of pot-honey, pot-pollen, cerumen, and propolis. Other pairs of keywords to be considered are phenolics and total.phenolic.content, flavonoids and total.flavonoid.content, proximal.composition and proximate.analysis. The names of the counties, cities, and villages are compulsory in the methods but possibly not useful in the keywords unless it is of pandemic origin. Even the names of the countries could be questioned as keywords, with either countries compulsory or banned for all, but documents with countries and others without seems rather confusing in keywords. These are topics for reflection in the search for a consensus. Should the ethnic name and the scientific name of the bee be a keyword or not? The biodiversity of stingless bees varies according to the objectives, only one can be included in the keywords but many would be impossible because permitted keywords are limited. Techniques and metabolites are also important keywords.

#### 5.1.7. Co-Authors’ Collaborative Networks and Country Collaborative Map in Pot-Pollen Research

**Figure 6:**
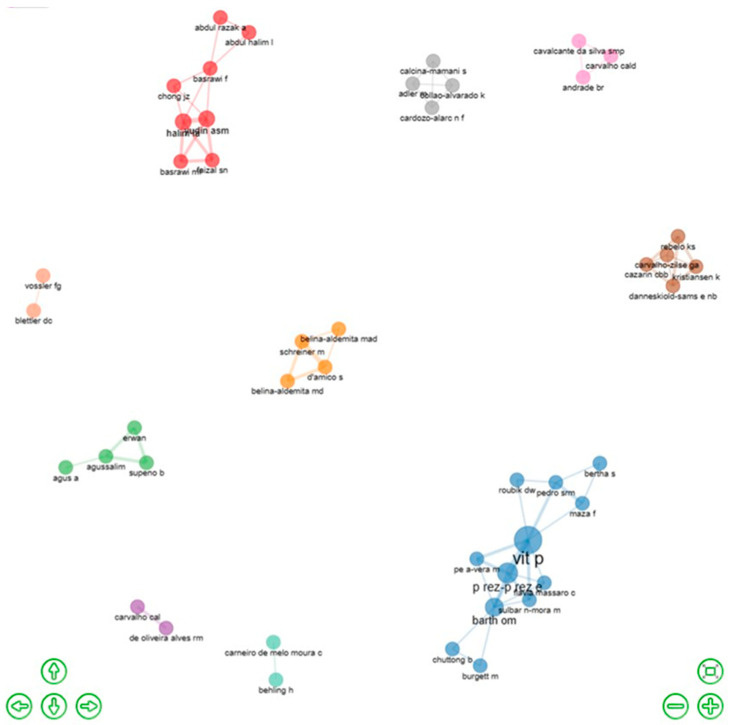
Collaborative networking of pot-pollen researchers since 2014.

The plot below shows the scientific teams of the pot-pollen documents in the dataset. The collaborative networks of co-authors were grouped in the 12 clusters of research groups, as identified in Figure 6. These comprise cluster 1 (red) with eight co-authors from Malaysia (halim la, yudin asm, basrawi f, basrawi mf, faizal sn, abdul halim l, abdul razak a, and chong jz), cluster 2 (pale blue) with eleven co-authors from Venezuela, Brazil, Australia, Ecuador, Panama, United States, and Thailand (vit p, barth om, perez-perez e, pedro srm, flavia massaro c, maza f, pena-vera m, roubik dw, sulbaran-mora m, bertha s, burgett m, and chuttong b), cluster 3 (green) with four co-authors from Indonesia (agussalim, erwan, supeno b, and agus a), cluster 4 (violet) with two co-authors from Brazil (carvalho cal, and de oliveira alves rm), cluster 5 (orange) with four co-authors from Austria and the Philippines (d’amico s, schreiner m, belina-aldemita md, and belina-aldemita mad), cluster 6 (brown) with five co-authors from Brazil, Denmark, and China (cazarin cbb, danneskiold-sams e nb, kristiansen k, rebelo ks, and carvalho-zilse ga), cluster 7 (pink) with three co-authors from Brazil and Portugal (andrade br, carvalho cald, and cavalcante da silva smp), cluster 8 (gray) with four co-authors from Bolivia (adler m, calcina-mamani s, cardozo-alarc nf, and collao-alvarado k), cluster 9 (turquoise) with two co-authors from Germany (behling h, carneiro de melo moura c), and cluster 10 (peach) with two co-authors from Argentina (vossler fg, and blettler dc). Ten interactive teams of 45 total researchers from 17 countries were visualized in the productive network of this plot. Six national teams were from Malaysia (cluster 1), Indonesia (cluster 3), Brazil (cluster 4), Bolivia (cluster 8), Germany (cluster 9), and Argentina (cluster 10). Four multinational teams with co-authors of at least two countries were from cluster 2 (seven countries), cluster 5 (two countries), cluster 6 (three countries), and cluster 7 (two countries).

**Figure 7:**
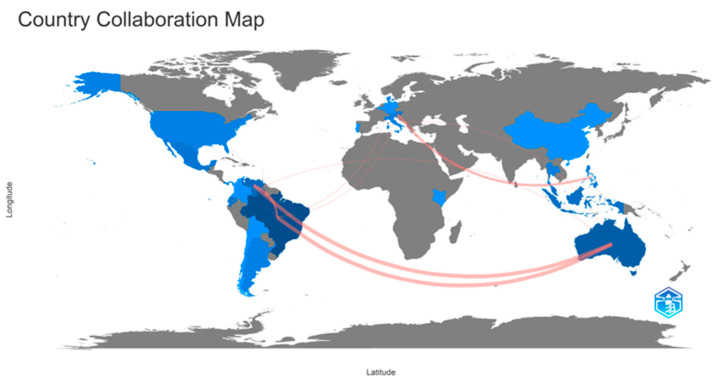
Worldwide map with country collaboration for pot-pollen research since 2014. Higher productivity is for dark blue than light blue countries. Collaborative rates are represented by red lines. Connecting countries have increasing line thickness with most frequently shared publications.

The pot-pollen research collaboration between countries was visualized with red connectors in a worldwide map between countries sharing publications on pot-pollen since 2014 in Figure 6. The frequencies of collaboration between two countries are available in the corresponding Excel file. The highest collaborative frequency (5) was between Brazil and Australia and Venezuela and Australia; (3) between Austria and the Philippines; and (2) between Australia and Ecuador, Australia and Italy, Brazil and Denmark, Brazil and Ecuador, and Brazil and Italy. Red lines between countries were plotted for two or more shared documents. Countries sharing one publication were not connected with red lines in Figure 7. The two thickest connectors show that the two most prolific multinational research teams in the worldwide collaborative map were Brazil and Australia and Venezuela and Australia.

#### 5.1.8. Conceptual Structure for Highest Contributions in Pot-Pollen Research

**Figure 8:**
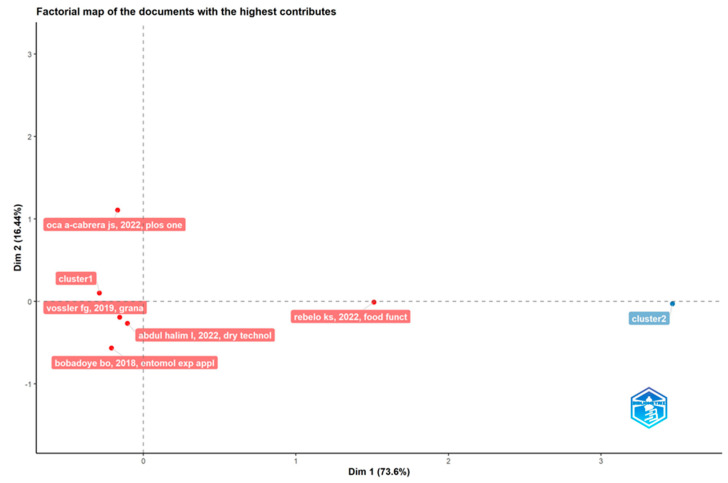
Factorial map of the pot-pollen documents with the highest contributions.

This section presents a plot of research impact. A black box is used to view the cluster components related with closeness calculated using the correspondence analysis (CA) multivariate statistical technique. Author keywords, the number of documents, and total citations per author are the factors considered for each dimension. In Figure 8, the pot-pollen documents with the most contributions after processing the dataset in a factorial map that generated one red cluster (2018–2022) is shown. None of these documents is a chapter in *Pot-pollen in stingless bee melittology*. Bobadoye et al.’s (2018) [24] *Entomological Experimental Applications*, Vossler et al.’s (2010) [103] *Grana*, Abdul Halim et al.’s (2022) [104] *Dry Technol*, Cerbulo-Vazquez et al.’s (2022) [105] *PlosOne*, and Rebelo et al.’s (2022) [91] *Food Function* are cited.

#### 5.1.9. Most Globally Cited Documents in Pot-Pollen Research

**Figure 9:**
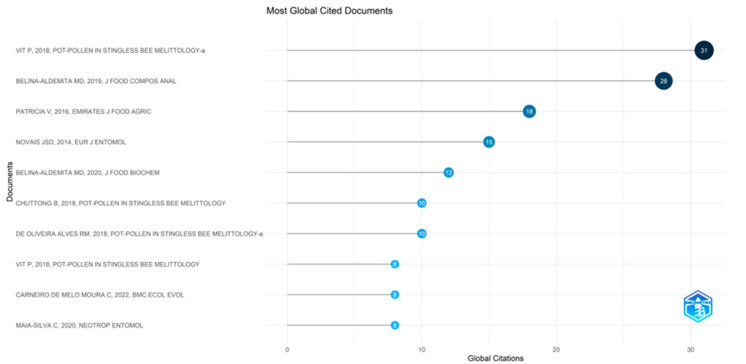
Most globally cited documents of pot-pollen from 2014 to 2023.

The plot of the most globally cited documents in Figure 9 shows the top ten documents cited from 8 to 31 times in publications of pot-pollen since 2014, with four of them published in the book on pot-pollen and the others in six diverse sources in a time span from 2014 to 2022. The different sizes of circles refer to the number of citations, and the darker blue shades have higher TC total counts per year. The most cited document is the pot-pollen book by vit p, 2018; it was cited 31 times and has 5.17 TC. The second most cited document is the article from belina-aldemita in 2019 in the *j food compos anal*, with 28 citations and 5.60 TC, a seminal contribution for the Philippine *Tetragonula biroi* pot-pollen chemical quality criteria. The third most cited is the article by patricia v in 2016 in *emirates j food agric*, with 18 citations and 2.25 TC for the proximate analysis, total flavonoid content, total polyphenol content, and total antioxidant activity of pot-pollen of two Venezuelan stingless bee species, *Melipona* aff. *eburnea* and *Scaptotrigona* sp. *ochrotricha*, of the Amazonian forest. Note that the Scopus database cannot correct the author’s surname and initial of Patricia V for the correct Vit P until the *Emirates Journal of Food and Agriculture* removes their editorial error, consequently reflected in the dataset used for this Bibliometrix plot.

### 5.2. Bibliometrics on Direct Injection in Food Flavor 

The dataset retrieved from the Scopus database consisted of 48 documents, with 44 in English, three in Chinese, and one in Afrikaans. The bibliometric search and top ten rankings were performed using the Scopus database in the “TITLE-ABS-KEY” field on 18 December 2023. The operator AND of all documents on direct injection AND food and the operator OR for the American and British words flavor OR flavour were used in the following query string:

TITLE-ABS-KEY (direct injection AND food) AND flavor OR flavour).

Further query strings were the following:

TITLE-ABS-KEY direct injection AND pollen AND (flavor OR flavour)—no documents; TITLE-ABS-KEY direct injection AND bee-bread AND (flavor OR flavour)—no documents.

TITLE-ABS-KEY direct injection AND pot-pollen AND (flavor OR flavour)—no documents.

The main information of bibliometric descriptors is presented in Table 12, including publications from the first retrieved document, from 1976 to 2023.

#### 5.2.1. Most Productive Authors on Direct Injection in Food Flavor

The top ten authors most productive in direct injection in food flavor shown in Table 13 are from Italy (8), Austria (1), and France (1), with eight to three documents in the period of 1976–2023.

#### 5.2.2. Geographical Distribution of Productive Institutions and Countries on Direct Injection in Food Flavor

In Table 14, the top ten institutions are from France (5), Italy (3), Austria (1), and the United States (1), which were ranked from eight to two documents published since 1976.

The ten most productive countries in direct injection in food flavor analysis research were ranked in Table 15. The top ten countries in direct injection in flavor analysis research produced ten to two documents each. The top five countries were from north America, Europe, and Asia, comprising the United States, Italy, France, China, and Spain. Austria, the Netherlands, Belgium, Canada, and India were included in the next five countries.

#### 5.2.3. Most Frequently Used Sources for Dissemination of Research on Direct Injection in Food Flavor

Table 16 shows the top ten sources used by authors to publish their research on direct injection in food flavor research from 1976 to 2023. Each of the listed journals hosted between four to one document each. The top five most productive sources were the *Journal of Agricultural and Food Chemistry*, *Food Research International*, *Journal of Mass Spectrometry*, *Analytical and Bioanalytical Chemistry*, and *Food and Fermentation Industries*. The journals’ h index varied between 328 and 71; 5/10 journals are in Quartile 1. The maximum impact score was 13.53 for the journal *Trac Trends in Analytical Chemistry*, ranked in the eight position in the dataset.

#### 5.2.4. Main Funding Sources on Direct Injection in Food Flavor

The funding sponsors supporting direct injection in food flavor analysis research are ranked in Table 17 according to the number of publications. Four of the top ten funding agencies that sponsored direct injection in food flavor research were from the European Union, three from the United States, and one each from China, France, and India.

#### 5.2.5. Main Subject Areas of Research on Direct Injection in Food Flavor

Table 18 shows that the top ten Scopus subject areas on direct injection in food flavor research since 1976 were mostly in agricultural and biological sciences (26.2%) and chemistry (26.2%). The following Scopus subject areas of interest were biochemistry, genetics, and molecular biology (21.4%), immunology and microbiology (6.0%), chemical engineering (4.8%), pharmacology, toxicology, and pharmaceutics (4.8%), neuroscience (4.8%), and 1.2% for engineering, environmental science, and materials science.

#### 5.2.6. Authors’ Keywords and Keywords Plus: Most Relevant Words and Dendrogram on Direct Injection in Food Flavor

**Figure 10:**
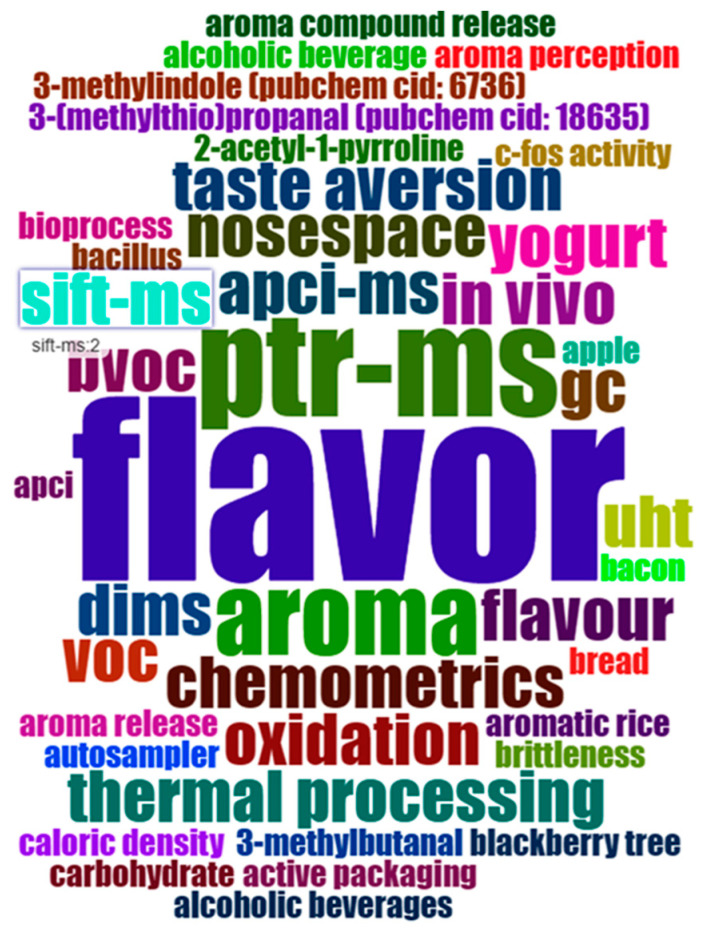
Word cloud by author keywords in the Scopus dataset of direct injection food flavor from 1976 to 2023.

In Figure 10, a word cloud plot was based on 50 author keywords in documents of the direct injection food flavor dataset from 1976 to 2023, with a frequency order from seven to one. The frequencies of author keywords correspond to different colors, position, and size. The largest and most central keywords are the most frequent and were visualized in this plot for flavor (7), ptr-ms (4), aroma (3), volatile compounds (3), apci-ms (2), bvoc (2), chemometrics (2), dims (2), direct-injection mass spectrometry (2), direct-injection mass spectrometry (dims) (2), flavour (2), gc (2), in vivo (2), and others.

**Figure 11:**
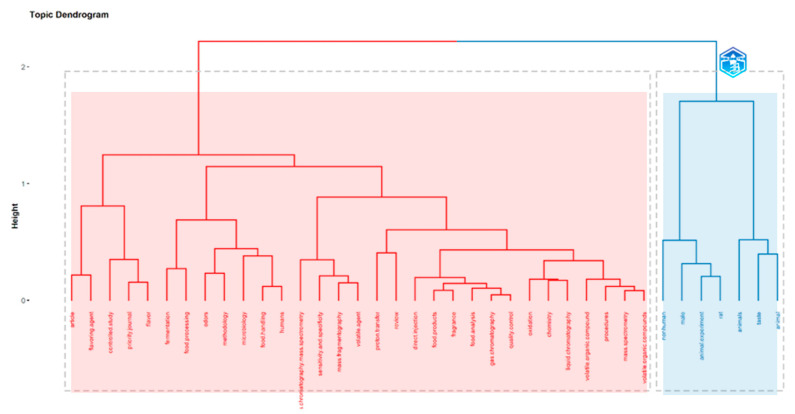
Topic dendrogram by HCA of keywords Plus in direct injection in food flavor publications from 1976 to 2023. The large red cluster has four branches, grouping topics on techniques including the following: direct injection, gas.cromatography, proton.transfer and review, fermentation, quality control, and volatile.organic.compounds. For the smaller blue cluster, animal-related words and taste are included.

In Figure 11, a dendrogram was built using hierarchical cluster analysis (HCA). Note that the keywords’ style of Bibliometrix is lowercase and italics are not used. Two clusters were observed, a large red cluster and a small blue cluster. Four branches of the red cluster 1 grouped the following keywords: 1.1 Flavouring.agent, controlled study, and flavor; 1.2 fermentation, food.processing, odors, methodology, microbiology, food.handling; 1.3 chromatography.mass.spectrometry, sensitivity.and.specificity, mass.fragmatography, and volatile.agent; 1.4a proton-transfer and review; 1.4b direct.injection, food.products, fragrance, food.analysis, gas.chromatography, and quality.control; 1.4c oxidation, chemistry, and liquid chromatography; 1.4d volatile.organic.compound, procedures, mass.spectrometry, and volatile.organic.compounds. For the smaller blue cluster 2, just animal-related words and taste were included.

#### 5.2.7. Co-Authors’ Collaborative Networks and Country Collaborative Map on Direct Injection in Food Flavor

**Figure 12:**
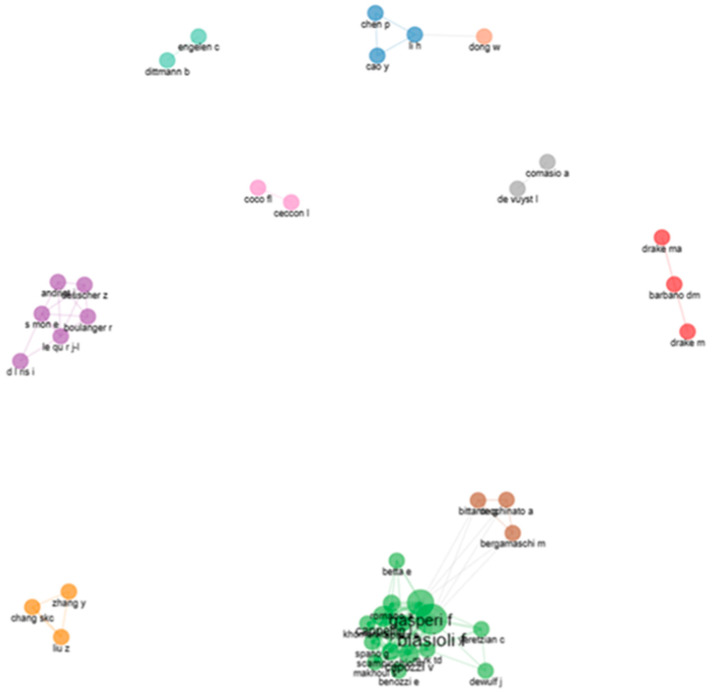
Collaborative networking of direct food flavor researchers from 1976 to 2023.

The plot below shows the scientific teams of the direct injection food flavor documents in the dataset. The collaborative networks of co-authors were grouped in the 10 clusters of research groups identified in Figure 12. These included cluster 1 (red) with three co-authors from the United States (barbano dm, drake m, and drake ma); cluster 2 (blue) with three co-authors from China (li h, cao y, and chen p); cluster 3 (green), the largest cluster, with fifteen co-authors from Italy, Belgium, France, Lebanon, and Switzerland (biasioli f, gasperi f, capozzi v, cappellin l, aprea e, khomenko I, m rk td, romano a, scampicchio m, makhoul s, spano g, yeretzian c, benozzi e, betta e, and dewulf j); cluster 4 (violet) with six co-authors from France (le qu r j-l, s mon e, andriot I, boulanger r, d l ris I, and deuscher z); cluster 5 (orange) with three co-authors from China (zhang y, chang skc, and liu z); cluster 6 (brown) with three co-authors from Italy (bergamaschi m, bittante g, and cecchinato a); cluster 7 (pink) with two co-authors from Italy (ceccon l and coco fl); cluster 8 (gray) with two co-authors from Belgium (comasio a and de vuyst l); cluster 9 (acquamarine) with two co-authors from Germany and the Netherlands (dittmann b and engelen c); and a lone node 10 (peach) from China for dong w.

Ten interactive teams of 40 total researchers from 10 countries were visualized in the productive network of this plot. Eight national teams were from the United States (cluster 1), China (cluster 2), France (cluster 4), China (cluster 5), Italy (cluster 6), Italy (cluster 7), Belgium (cluster 8), and China (lone node 10). Two multinational teams with co-authors of at least two countries were from cluster 3 (Italy, Belgium, France, Lebanon, and Switzerland) and cluster 9 (Germany and the Netherlands).

**Figure 13:**
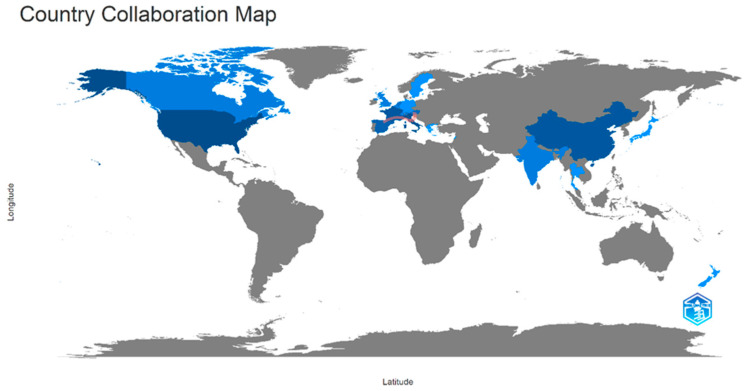
Worldwide map with country collaboration for direct injection food flavor research from 1976 to 2023. Dark blue countries are more productive than light blue countries. Collaborative rates represented by red lines between countries are visualized between Italy and Austria (4) and Italy and France (3). Connecting countries with increasing line thickness have most frequently shared publications.

The direct injection food flavor research collaboration between countries was visualized with red connectors between Italy and Austria with four shared documents and Italy and France with three shared documents in the worldwide map between countries sharing publications from 1976 to 2023, shown in Figure 13. The frequencies of collaboration between two countries are available in the corresponding Excel file. Frequencies lower than three are not visualized in the country collaboration map, for example, between Austria and Switzerland (2) and Italy and Switzerland (2). Countries sharing one document are Austria and Belgium, Belgium and Switzerland, China and the United States, France and Austria, France and Lebanon, Italy and Belgium, Italy and Lebanon, Italy and New Zealand, Germany and the Netherlands, Sweden and the United States, and Thailand and the United States.

#### 5.2.8. Conceptual Structure for Highest Contributions on Direct Injection in Food Flavor

**Figure 14:**
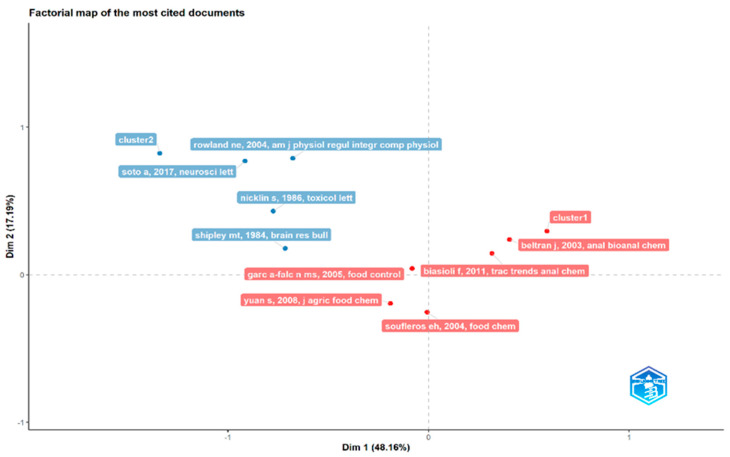
Factorial map of the most cited direct injection food flavor documents from 1976 to 2023.

In the plot of research impact in Figure 14, the direct injection food flavor documents with the most contributions were, after processing the dataset in a factorial map, a red cluster 1 with five documents (2003–2011) and a blue cluster 2 with four documents (1984–2017). A conceptual structure matches different ideas on a topic that are linked together. The finding from the conceptual structure is the research domains by dimensions or factors like keywords, no. of documents per authors, and TC. There are two clusters across two dimensions or factors. The first dimension, Dim 1, explained 48.16% of the variation, and the second, Dim 2, explained 27.19%. Two documents belonging to cluster 1 are positioned in the positive quadrants of both dimensions (beltran j, 2003 and biasioli f, 2011).

This factorial map of the most cited documents has cluster 1 (red) grouping, according to beltran j, 2003, *anal bioanal chem*; soufleros eh, 2004, *food chem*; garc a falc n ms, 2005, *food control*; yuan s, 2008, *j agr food chem*; and biasioli f, 2011, *trac trends anal chem*. Cluster 2 (blue) grouping was determined according to shipley mt, 1984, *brain res bull*; nicklin s, 1986, *toxicol lett*; rowland ne, 2004, *am j physiol regul integr regul physiol*; and soto a, 2017, *neurosci lett*.

#### 5.2.9. Most Globally Cited Documents on Direct Injection in Food Flavor

**Figure 15:**
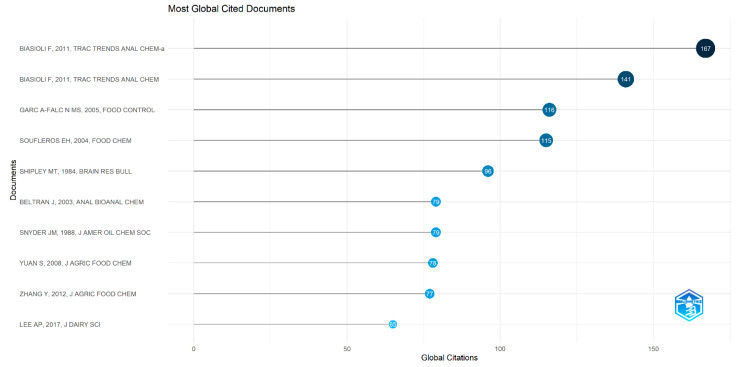
Most globally cited documents of direct injection in food flavor from 1976 to 2023.

The plot of most globally cited documents in Figure 15 shows the top ten documents cited from 167 to 78 times in publications of direct injection in food flavor from 1976 to 2023. The most cited were by teams led by Biasioli F, 2011, *Trac Trends Anal Chem*-a (167 citations), Biasioli F, 2011, *Trac Trends Anal Chem* (141), Garc A-Falc N MS, 2005, *Food Control* (116), Soufleros EH, 2004, *Food Chem* (115), Shipley MT, 1984, *Brain Res Bull* (96), Beltran J, 2003, *Anal Bioanal Chem* (79), Snyder JM, 1988, *J Amer Oil Chem Soc* (79), and Yuan S, 2008, *J Agric Food Chem* (78). Two of them were published in the *Trac Trends Anal Chem* and the others in eight diverse sources in a time span from 1984 to 2011. Different circle sizes refer to the number of citations, and the darker blue shades have higher TC total counts per year. The most cited document is by biasioli f, 2011, which is a comprehensive review that was cited 167 times and has 12.85 TC, titled Direct-injection mass spectrometry adds the time dimension to (B)VOC analysis. The second most cited document is another article by biasioli f, 2011, with 141 citations and 10.85 TC on the emerging technique of PTR-ToF-MS for the direct, fast, and sensitive monitoring of volatile organic compounds (VOCs) and biogenic VOCs (BVOCs) addressed in food science. The third most cited document is the article by Garc A-Falc N MS, 2005, in *Food Control*, with 116 citations and 6.11 TC. Her surname is García-Falcón, but Bibliometrix deletes letters with accents and the rest. In this research, five synthetic food colors were added to soft drinks to optimize a method to determine food dyes.

### 5.3. Comparative Metrics Between Pot-Pollen and Direct Injection Food Flavor Publications

As seen in Table 19, pot-pollen is a more recent topic of interest than direct injection food flavor, with the first documents in the Scopus database in 2016 and 1976, respectively. Double the number of articles was published for direct injection than pot-pollen datasets, the latter standing out for more book chapters. The annual growth rate is half in direct injection compared to pot-pollen. Direct injection disseminated research findings are in almost double the source types than pot-pollen. The average age is more than threefold in direct injection than pot-pollen. The average number of citations per document of direct injection are six times greater than citations of pot-pollen. International co-authorships are duplicated in pot-honey documents compared to direct injection. The average number of co-authors per document and number of references are similar in both datasets.

Looking at the Scopus ranking tables, some similarities and differences are clearly showing scientific interests and support; for example, pot-pollen research has four funding sponsors from Brazil, whereas four European Union and three United States funding agencies sponsored direct injection in food flavor. This fact makes a substantial difference in the development of these disciplines, setting Europe and the United States as geographical supremacies for technological DIFFA productive and innovative applications. Brazil remains the most biodiverse country on stingless bee richness, with 359/605 global species [106] and the largest pot-pollen production, named Samburá in Brazil [20]. However, Austria and Denmark are also in the top ten funding sponsors, which is important for European interaction in multinational projects on shared research interests on a tropical nest material investigated with DIFFA for potential health and environmental discoveries. Similarly, the most productive countries are Neotropical (Brazil, Venezuela, Mexico, Ecuador, and Argentina) and paleotropical (Australia, Indonesia, Malaysia, Philippines), with Austria representing Europe for pot-pollen research and Europe (Italy, France, Spain, Austria, the Netherlands, and Belgium), North America (United States and Canada), and Asia (China and India) for DIFFA.

The top ten authors have similar productivity, and both pot-pollen (six to two docs) and direct injections in food flavor (eight to three docs) are growing disciplines. This is similar for the institutions leading pot-pollen (7–3 docs) and direct injection food flavor (8–2 docs) research. Agricultural and biological sciences is the top subject area and biochemistry, genetics, and molecular biology for pot-pollen and direct injection in food flavors. Additionally, chemistry for direct injection techniques and engineering for dehydration studies are needed to extend pot-pollen shelf life. The journals used by both topics of research are different; higher h index, quartiles, and impact factors are observed for the top ten DIFFA than pot-pollen sources.

## 6. Pot-Pollen, a Biodiverse Product with a Geographical, Entomological, Botanical, and Less Studied Microbial Origin

Some taxa have more similar pot-pollen than others among the 605 stingless bee species [1]. The first multivariate study permitted partial entomological separations of 25 samples of pot-pollen of the following six species of stingless bees from Brazil: 1. *Melipona scutellaris*; 2. *Melipona subnitida*; 3. *Melipona quadrifasciata anthidioides*; 4. *Plebeia* sp.; 5. *Tetragona clavipes*; and 6. *Melipona asilvai*. The *Tetragona clavipes* cluster differentiated from *Melipona scutellaris*, but environmental factors may have influenced unexpected similarities. For example, sharing botanical resources may explain the *Plebeia* sp. scattered in diverse clusters [107].

The foraging habits of floral pollen by diverse stingless bee species are studied with palynological techniques, either natural pollen or after acetolysis [28,108]. Corbicula pollen and pot-pollen are analyzed to identify the botanical origin of polleniferous plants visited by stingless bees. Morphological features of pollen grains are observed using a light microscope to describe variable pollen size from about 10 to 300 µm; a variety of spheric, oblate, spheric or prolate shapes; smooth to ornamented granular, grooved, spined, or striated surfaces; the aperture number and aperture type of porate, colpate, or colporate; and single or polyad arrays, all characteristics noted by palynologists. Comparisons with reference pollen collections and pollen atlases, such as by Barth (1989) [29], are the matching fingerprints of botanical origin at the species, genus, or family level.

## 7. Conclusions

Pot-pollen unique chemical composition and medicinal uses positions a new health-oriented bee product for consumers in the market, a candidate for pharmaceutical development like the *Apis mellifera* bee bread. A chemical suite of more than a hundred VOCs characterized Australian and Venezuelan pot-pollen biomolecules in this review. The critical assessment on increasing global antimicrobial resistance by the World Health Organization demands policy frameworks providing insights from microbiology, pharmacology, and innovative solutions where pot-pollen can play a role in synergistic efficacy with conventional antibiotics.

The first document retrieved in the bibliometric search of pot-pollen with the Scopus database was on palynology in 2014, evidence of a recent scientific interest, developing further chemical and bioactive characterizations of this medicinal food. The book *Pot-Pollen in Stingless Bee Melittology* hosted 16/40 documents. Pot-pollen was in the title of 14/15 of the book chapters and in 14 other documents published after 2018. Compared with pot-honey and propolis, pot-pollen is a lesser studied product of the stingless bee nest, with functional properties attracting multidisciplinary bee scientists. Our modest dataset of 40 documents (2014–2023) covered the chemical composition such as proximate analyses, macronutrients, minerals, primary metabolites, secondary metabolites, and volatile organic compounds; biological activity and microbiology; nutritional and therapeutic effects in silico, in vitro, and in vivo using animal models; palynological applications to the botanical origin, bee diet, and reforestation; conservation methods of dehydration, a proposal of standards, good practices of hygiene, and indicators of productivity of pot-pollen were reviewed for 37 Neotropical, 11 Indo-Malaysian, and three Australian species of stingless bees.

The first document on direct injection in food flavor research retrieved with the Scopus database was on undesirable effects on the flavor and nutritive value of pasteurized milk by ultra-high temperature (UHT) in 1976 in a set of 48 documents (1976–2023) covering the comparison between SPME-GC-MS and PTR-ToF-MS analysis, direct-injection mass spectrometry for a VOC analysis of flavor dosage discrimination, flavor enhancement, flavor perception, a high-throughput method to test flavor-forming, PTR-ToF-MS for bioprocess monitoring and nose-space analysis, the removal of flavors, screening strains for direct vat set fermentation, shelf-life solid-phase microextraction, and VOCs of commercial bakery yeasts of wine and beer origin. Pot-pollen VOC data were partly presented at DIFFA23 (Direct Injection Food Flavor Analytics), shown by Betta et al. (2023) [26], the first edition of an international symposium on methodologies for the analysis of volatile compounds using direct methods, focusing particularly on direct-injection mass spectrometry.

## 8. Future Directions

This traditional stingless bee product is a new material for scientific research recently started and slowly growing in publications. Pot-pollen has potential development for therapeutic and pharmaceutical applications. The microbial associations with the 605 stingless bee species and techniques of direct injection in food science with HS-SPME-MS or PTR-ToF-MS are promising new discoveries in stingless bee nest volatiles, including pot-pollen. Multi-country authored documents are needed for vital cooperation between tropical habitats where pot-pollen is produced and specialized non-tropical laboratories devoted to bioanalytical techniques, with experts on direct injection in food flavor analysis (DIFFA) at Foundation Edmund Mach in Italy with a solid background to explore new food materials and the bio-transformations caused by fermentation. After this first review on pot-pollen VOCs, more questions than answers were expected. The pot-pollen microbiome is an important demonstration to explain a portion of the functional VOCs observed, the microbial richness, how it is affected by the environment, seasons, botanical origins in the same or diverse stingless bee species, and even by climate change. These are questions with no immediate responses. The bouquet of the stingless bee nest has not been studied and it is as fragrant as wine or cheese. Distinctive VOCs for each of the 605 species of stingless bee nests would require an approach for elucidating their ecology, social immunity, and perhaps an answer on why *Varroa* prefers *Apis mellifera*. Can stingless bee cerumen or propolis extracts be *Varroa* deterrents in honeybee colonies? Or would it alter the honeybee equilibrium? VOCs with functions for the stingless bee colony are useful for medicinal therapies and pharmaceutical applications.

No evidence of adverse effects of *Eucalyptus* honeybees or pot-pollen were found. Good practices of stingless beekeeping and the creation of new standards for pot-pollen norms will benefit consumers, industrial developments, and stingless beekeepers. Besides the microbial origin of beneficial metabolites of bee bread, mycotoxins of fungal origin have been reported [109]. Therefore, safety studies like those initiated by Belina-Aldemita et al. (2020) [53] will be needed to protect producers and consumers.

Integrative direct injection in pot-pollen flavor would be a novel approach demanding experimental protocols at the stingless bee nest scale—simulating those initiated with other food by Capozzi et al. (2017) [27] on applications to quantitate VOCs in the following main scenarios: 1. on-line bioprocess monitoring of VOCs released during lactic acid fermentation in stingless bee nest biotechnology; 2. screening pot-pollen VOCs for each stingless bee species considering botanical, seasonal, and habitat origins; 3. identification of distinctive VOCs and their bio-transformations into derived biomolecules produced by microbial associations with stingless bee species; 4. retronasal VOC release during pot-pollen tasting alone or lemonade-like preparation by nose-space analysis; and 5. creation of a multidisciplinary team to boost direct injection in pot-pollen flavor for nutraceutical, medicinal, and pharmaceutical developments.

We propose a book on *Direct injection in pot-pollen flavor* as a result of careful insights after the bibliometric reviews on pot-pollen and direct injection in food flavor analysis and the initial VOCs of pot-pollen produced by Australian and Venezuelan stingless bees from Table 1. This starter of direct injection HS-SPME-GC/MS in pot-pollen deserves expansion and comprehensive continuation, including PTR-ToF-MS. All materials of the stingless bee nest would solve a puzzle but need wise choices for an otherwise endless endeavor. Sixty years later, the historical collaborative trend has not changed since Paulo Nogueira-Neto sent pot-honey from Brazil to France to learn that they have higher moisture and free acidity than *Apis mellifera* honey, described by Gonnet et al. (1964) [110] (as well as comparing *Apis mellifera* bee bread with pot-pollen (P. Vit, personal observation)). Symbiont microorganisms are primarily responsible for the fermentation that preserves pot-honey and pot-pollen in the storage area of the stingless bee nest [111,112]. In contrast, Apini uses dehydration strategies to preserve honey [111]. Particularly, *Apis mellifera* does not rely on fermented food but on more dehydrated food for the bee colony and thus processes honey and bee bread with lower moisture contents than Meliponini pot-honey and pot-pollen.

A driving force towards industrial production would possibly limit DIFFA availability to ancestral medicinal uses of tropical fermented materials of the stingless bee nest, as well as physiological and ecological functions of VOC suites. Additionally, a book is needed with chapters on the synergism of pot-pollen from diverse countries, vegetations, and stingless bee species, with conventional antibiotics to fill the gap in the emerging potential to overcome antimicrobial resistance. A tentative title expanding to pot-honey, cerumen, and propolis, is *Stingless Bee Nest Materials: Novel anti-antimicrobial-resistant agents.* However, it is a scientific challenge to meet the excellence of both territories, overcoming human, institutional, financial, customs, and especially cultural limitations that may prevent discoveries.

## Figures and Tables

**Table 1:** Volatile metabolites of stingless bee pollen of Australian *Austroplebeia australis*, *Tetragonula carbonaria*, and *Tetragonula hogkingsi* [41] and Venezuelan *Tetragonisca angustula* [26].

No. Australia	^1^ No. Venezuela	Chemical Classes of Volatile Organic Compounds (VOCs)	Presence of VOCs in Stingless Bee Species			
			Australia			Venezuela
			*Austroplebeia australis*	*Tetragonula carbonaria*	*Tetragonula hogkingsi*	*Tetragonisca angustula*
		**1. Acids (11)**				
**1**	**1**	acetic acid	-	+	+	+
	**2**	propanoic acid	-	-	-	+
	**3**	2-methyl propanoic acid	-	-	-	+
	**4**	butanoic acid	-	-	-	+
	**5**	3-methyl butanoic acid	-	-	-	+
	**6**	2-methyl butanoic acid	-	-	-	+
	**7**	3-methyl pentanoic acid	-	-	-	+
	**8**	pentanoic acid	-	-	-	+
	**9**	tiglic acid	-	-	-	+
	**10**	hexanoic acid	-	-	-	+
	**11**	octanoic acid	-	-	-	+
		**2. Alcohols (16)**				
	**12**	2-propanol	-	-	-	+
	**13**	ethanol	-	-	-	+
	**14**	2-methyl-3-buten-2-ol	-	-	-	+
	**15**	2-methyl-1-propanol	-	-	-	+
	**16**	2-pentanol	-	-	-	+
	**17**	1-butanol	-	-	-	+
	**18**	2-methyl butanol + 3-methyl butanol3	-	-	-	+
	**19**	1-pentanol	-	-	-	+
	**20**	2-heptanol	-	-	-	+
	**21**	hexanol	-	-	-	+
	**22**	3-octanol	-	-	-	+
	**23**	1-heptanol	-	-	-	+
	**24**	6-methyl-5-hepten-2-ol	-	-	-	+
	**25**	2-ethyl-1-hexanol	-	-	-	+
	**26**	benzyl alcohol	-	-	-	+
	**27**	2-phenylethanol	-	-	-	+
**2**		cis-geraniol	+	-	-	-
**3**		geraniol	+	+	+	-
**4**		benzemethanol	+	+	+	-
**5**		epiglobulol	-	+	+	-
**6**		(−)spathulenol	+	+	+	-
		**3. Aldehydes (7)**				
**7**	**28**	hexanal	+	+	+	+
	**29**	(Z)o(E)-2-heptenal	-	-	-	+
**8**	**30**	nonanal	+	+	-	+
	**31**	(E)-2-octenal	-	-	-	+
	**32**	furfural	-	-	-	+
	**33**	benzaldehyde	-	-	-	+
	**34**	benzeneacetaldehyde	-	-	-	+
**9**		p-anisaldehyde	+	+	+	-
**10**		α-citral	+	-	-	-
**11**		methanone	+	+	+	-
		**4. Esters (16)**				
	**35**	methyl acetate	-	-	-	+
	**36**	ethyl acetate	-	-	-	+
	**37**	isopropyl acetate	-	-	-	+
	**38**	butanoic acid ethyl ester	-	-	-	+
	**39**	butanoic acid-2-methyl ethyl ester	-	-	-	+
	**40**	ethyl isovalerate	-	-	-	+
	**41**	3-methyl butyl acetate	-	-	-	+
	**42**	hexanoic acid ethyl ester	-	-	-	+
	**43**	heptanoic acid ethyl ester	-	-	-	+
	**44**	ethyl (L)-(-)-lactate	-	-	-	+
	**45**	ethyl 3-hydroxybutanoate	-	-	-	+
	**46**	ethyl succinate	-	-	-	+
	**47**	ethyl benzene acetate	-	-	-	+
	**48**	2-phenethyl acetate	-	-	-	+
	**49**	delta octalactone	-	-	-	+
	**50**	5,6-dihydro-6-propyl-2H-pyran-2-one	-	-	-	+
		**5. Ketones (8)**				
	**51**	acetone	-	-	-	+
	**52**	2-butanone	-	-	-	+
	**53**	3-pentanone	-	-	-	+
	**54**	2-methyl-3-pentanone	-	-	-	+
	**55**	2-heptanone	-	-	-	+
	**56**	3-octanone	-	-	-	+
	**57**	6-methyl-5-hepten-2-one	-	-	-	+
	**58**	2-nonanone	-	-	-	+
**12**		4-ketoisophorone	+	-	-	-
**13**		methanone	+	+	+	-
		**6. Monoterpenes (17)**				
	**59**	1R-α-pinene	-	-	-	+
	**60**	α-thujene	-	-	-	+
	**61**	camphene	-	-	-	+
	**62**	β-pinene	-	-	-	+
	**63**	β-thujene	-	-	-	+
	**64**	3-carene	-	-	-	+
	**65**	α-phellandrene	-	-	-	+
	**66**	β-myrcene	-	-	-	+
	**67**	α-terpinene	-	-	-	+
	**68**	limonene	-	-	-	+
	**69**	eucalyptol	-	-	-	+
	**70**	β-phellandrene	-	-	-	+
	**71**	γ-terpinene	-	-	-	+
	**72**	linalool	-	-	-	+
	**73**	terpinen-4-ol	-	-	-	+
	**74**	α-terpineol	-	-	-	+
	**75**	borneol	-	-	-	+
**14**		α-pinene	+	+	+	-
		**7. Oxides (5)**				
	**76**	cis-linalool oxide	-	-	-	+
	**77**	trans-linalool oxide	-	-	-	+
	**78**	trans-pyranoid linalool oxide	-	-	-	+
	**79**	cis-pyranoid linalool oxide	-	-	-	+
**15**	**80**	caryophyllene oxide	+	+	+	+
		**8. Sesquiterpenes (11)**				
	**81**	α-cubebene	-	-	-	+
**16**	**82**	α-copaene	+	+	+	+
	**83**	β-bourbonene	-	-	-	+
	**84**	β-copaene	-	-	-	+
**17**	**85**	caryophyllene	-	+	+	+
	**86**	aromandendrene	-	-	-	+
	**87**	humulene	-	-	-	+
	**88**	γ-muurolene	-	-	-	+
	**89**	β-selinene	-	-	-	+
	**90**	γ-cadinene	-	-	-	+
	**91**	cedrol	-	-	-	+
**18**		sesquiterpene 1	+	+	+	-
**19**		sesquiterpene 2	+	+	+	-
**20**		sesquiterpene 3	+	+	+	-
**21**		sesquiterpene 4	+	+	+	-
**22**		alloaromadendrene	+	+	+	-
**23**		(+)-ledene	-	+	+	-
**24**		sesquiterpene 5	-	+	+	-
**25**		sesquiterpene 6	+	+	+	-
**26**		sesquiterpene 7	+	+	-	-
**27**		*cis*-α-bisabolene	+	+	-	-
		**9. Others (4)**				
	**92**	2,3-butanediol (polyol)	-	-	-	+
	**93**	propylen glycol (polyol)	-	-	-	+
	**94**	estragole (phenylpropene)	-	-	-	+
	**95**	glycerin (polyol)	-	-	-	+
**28**		labd-14-ene	-	+	+	-
		**10. Hydrocarbons**				
**29**		1H-cycloprop[e]azulene	+	+	+	-
**30**		hydrocarbon 1	+	+	+	-
**31**		hydrocarbon 2	+	+	+	-
**32**		hydrocarbon 3	-	+	+	-
**33**		hydrocarbon 4	+	+	+	-
**34**		hydrocarbon 5	+	+	+	-
**35**		hydrocarbon 6	+	+	+	-
		**Total**	28	32	29	95

^1^ Metabolites were numbered according to their linear retention indices, LRIs, calculated on the HP-INNOWax capillary column. (-) absence (+) presences.

**Table 2:** Descriptive olfactory attributes of pot-pollen VOCs produced in stingless bee nests from Australia and Venezuela (See Table 1).

Metabolites Alphabetical Order	Chemical Formula	Chemical Structures	Origin Food, Microorganisms, Plants	Olfactory Attributes
Acetic acid	CH_3_COOH	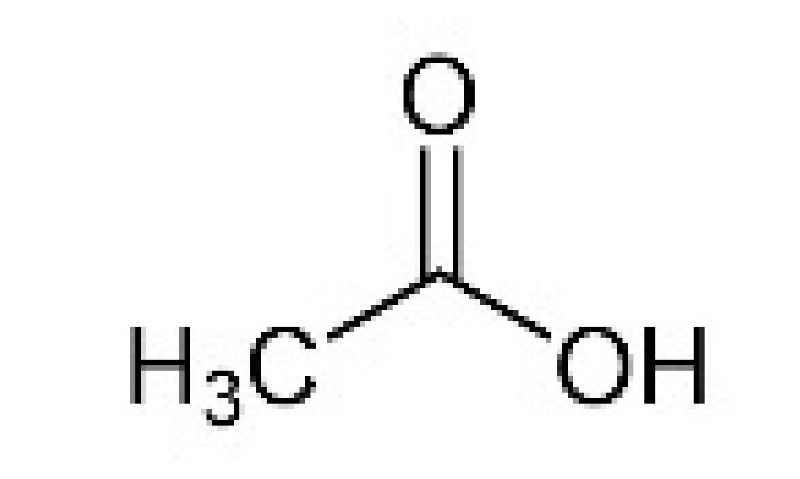	Acetic acid bacteria	Pungent smell, vinegar
*p*-Anisaldehyde	C_8_H_8_O_8_	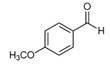	*Vanilla pompona, Solidago odora, Nigella sativa*	Anise-like
2,3-Butanediol	C_4_H_10_O_2_	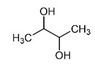	*Bacillus* spp.	Neutral, wine
Butanoic acid	C_4_H_8_O_2_	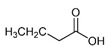	Butter ghee milk	Rancid butter
Ethanol	CH_3_CH_2_OH	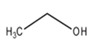	Yeasts	Distilled-like but not any particular rum, tequila, vodka, wine
Ethyl acetate	C_4_H_8_O_2_	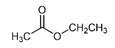	*Vitis rotundifolia,* *Cinnamomum sieboldii*	Pleasant, sweet, fruity
Furfural	C_4_H_3_OCHO	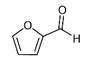	Chemical transformation	Almond-like
Glycerin	C_3_H_8_O_3_	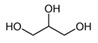	Beer honey vinegar wine	Odorless
Limonene	C_10_H_16_	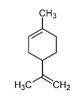	Oils of grapefruit, lemon, and orange	Lemon-like, citrusy
3-Methyl butanoic acid	C_5_H_10_O_2_	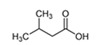	*Valeriana officinalis*	Rancid, cheesy, sweaty
3-Methyl butyl acetate	C_7_H_14_O_2_	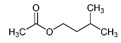	*Vitis rotundifolia, Nicotiana bonariensis*	Artificial banana
2-Methyl-1-propanol	C_4_H_10_O	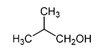	Fresh tea leaves	Sweet, musty
β-Phellandrene	C_10_H_16_	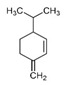	Chemical transformation, Canada balsam oil	Peppery, minty, citrusy
2-Phenylethanol	C_8_H_10_O	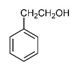	*Vitis rotundifolia, Lonicera japonica, Moringa oleifera*	Floral
α-Pinene	C_10_H_16_	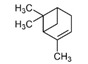	Eucalyptus oil, pine trees, rosemary	(+)-α-Pinene minty, (−)-α-Pinene pine, turpentine
Propylene glycol	C_3_H_8_O_2_	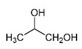	Soybean and canola oils	Odorless

**Table 3:** Biological activity of most abundant volatile metabolites of *Tetragonisca angustula* pot-pollen from Merida, Venezuela.

Metabolites Descending Order of Abundance	Chemical Structures	Metabolite Abundance	Origin M, Microbial P, Plant C, Chemical Transformation	Biological Activity (Reference)
Acetic acid	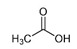	50.41	M	Antibacterial activity in diluting solutions against *P. aeruginosa* [55,56]
2,3-Butanediol	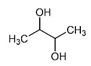	23.88	M	Reduction in oxidative stress, anti-inflammatory, and antihypertensive properties [57,58,59]
β-Phellandrene	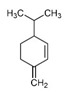	9.73	P	Anticancer and antibacterial activity [60,61]
Propylene glycol	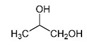	7.08	M	Antibacterial activity against *S. aureus* and *P. aeruginosa* [62,63]
2-Methyl-1-propanol	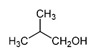	4.47	M	Antibacterial activity against *S. aureus* [64,65]
Furfural	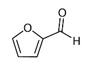	3.70	C	Antibacterial, antioxidant, and anti-inflammatory activity [66,67,68]
Ethanol	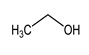	3.32	M	Effect of ethanol concentrations in antimicrobial activity of extracts [69]; extraction solvent used to prepare herbal remedies, a cutaneous penetration enhancer, and disinfecting and sterilizing agent
Ethyl acetate	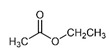	2.44	M, P	More than 5% ethyl acetate is antimicrobial [70]; extraction solvent of analytes in complex biological matrices
Glycerin	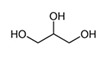	1.82	M, P	Antibacterial activity against *Streptococcus mutans*, *Staphylococcus* *aureus*, *Enterococcus* *faecalis*, and *Escherichia coli* [62]; sweetener in syrups and excipient in eyewash solutions
Butanoic acid	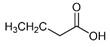	2.52	M	Mucosal health and energy source for colon cells [71]; additive to increase fruit fragrance
2-Phenylethanol	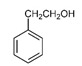	2.85	M, P	Broad-spectrum antifungal activity by suppressing mycelium growth, structural damage to mycelia, ROS stress, and cell membrane disruption [72]; antimicrobial, antiseptic, and disinfectant that is used also as aromatic essence and preservative
3-Methyl butanoic acid	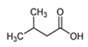	4.74	M, P	Antifungal activity against spore germination and mycelial growth of *Colletotrichum gloeosporioides* emitted by *Bacillus velezensis* CE 100 [73]; antifibrotic agent to treat scleroderma as an antirheumatic drug; flavoring cheese
Limonene	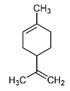	2.48	P	Broad-spectrum and long-lasting resistance to pathogen infection—insecticide, antifungal, anti-viral, and plant immunity activator [74]; promotes weight loss, prevents cancer, treats cancer, and treats bronchitis; cutaneous penetration enhancer for medicinal ointments and creams
5,6-Dihydro-6-propyl-2H-pyran-2-one	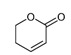	1.59	P	Anxiolytic, antidepressant, anti-inflammatory, thrombolytic, and cytotoxic activities [75]; treating benign prostatic hypertrophy or hyperplasia, prostatic cancer, alopecia, hirsutism, and acne vulgaris
3-Methyl butyl acetate	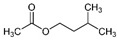	1.67	P	3-methyl butyl acetate has main anticancer role [76]; masking agent, perfuming agent, and solvent in cosmetics industry

After [26].

**Table 4:** Biological activity of selected volatile metabolites of *Austroplebeia australis*, *Tetragonula carbonaria*, and *Tetragonula hockingsi* pot-pollen from Queensland, Australia.

Metabolites Ascending Linear Retention Indices	Chemical Structures	Metabolite Abundance			Origin M, Microbial P, Plant	Biological Activity (Reference)
		Stingless Bees ^1^				
		Aa	Tc	Th		
α-Pinene	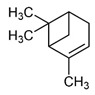	1.6	4.4	1.5	P	The anticancer effect of α-pinene was mediated by natural killer (NK) cell activation and cytotoxicity via extracellular signal-regulated kinase/protein kinase B (ERK/AKT) signaling pathways [77], Anti-inflammatory properties, antimicrobial properties, antiulcerogenic and gastroprotective properties, and the ability to aid memory are
*p*-Anisaldehyde	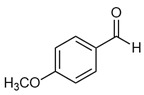	6.7	0.2	0.2	P	intermediate in the synthesis of other compounds important in pharmaceuticals and perfumery. Antimicrobial activity against *Pseudomonas aeruginosa* is via membrane transport, lipid biosynthesis, and stress response, as well as synergism with epigallocatechin gallate [78].
1H-Cycloprop[e]azulene	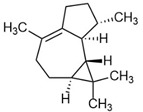	1.4	7.7	6.4	P	Biological activity in breast cancer cell lines towards estrogen receptors was found using the MTT test [79]. Azulene and its derivatives are antiallergic, antibacterial, and anti-inflammatory therapies.
*cis*-α-Bisabolene	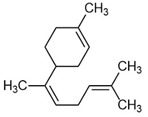	0.6	0.1	-	P	An antifungal agent for the plant has anticancer properties but β-bisabolene is more active against human and murine breast cancer cells [80]. It is used as an effective ligand with pregnane X receptor alternative to vitamin E and can penetrate the blood–brain barrier [81].
Epiglobulol	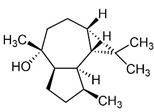	-	0.3	0.1	P	An anti-inflammatory sesquiterpene alcohol isolated from the bark of *Moringa oleifera* and *Eucalyptus* leaves [82].
Labd-14-ene-8	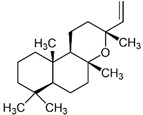	-	0.8	0.4	M, P	Presented in a review on sclareol human or murine cancer (cervical, breast, erythroleukemia, colon, gastric, lung epithelial) prevention and treatment [83]. A plant metabolite or a fungal metabolite, as well as an antibacterial agent.

^1^ The Australian stingless bees are **Aa:** *Austroplebeia australis*; **Tc:** *Trigona carbonaria*; and **Th:** *Trigona hockingsi*. After [41].

**Table 5:** Bibliometric descriptors of published research on pot-pollen since 2014.

Bibliometric Descriptor	Counts
	All Documents
Time span	2014–2023
Scopus database	
Number of documents	40
Number of articles	18
Number of books	1
Number of book chapters	15
Number of conference papers	4
Number of conference reviews	1
Number of reviews	1
Number of languages	3
*Bibliometrix*	
Annual growth rate (%)	8.01
Sources (no. journals, books)	20
Author’s keywords DE (no.)	73
Keywords Plus ID (no.)	170
Average citations per document	6.08
Document average age (years)	3.62
Authors (no.)	153
Single-authored documents (no.)	3
Multi-authored documents (no.)	38
International co-authorship (%)	45
Average co-authors per document (no.)	4.88
References (total no.)	1976

**Table 6:** Top ten most productive authors in pot-pollen research since 2014 with their affiliations and countries.

Ranking	NP ^1^	Pot-Pollen Research		
		Author	Affiliation, City	Country
**1**	6	Vit, P.	Food Science Departament, Faculty of Pharmacy and Bioanalysis, Universidad de Los Andes, Mérida	Venezuela
**2**	4	Barth, O.M.	Instituto Oswaldo Cruz, Fiocruz, Rio de Janeiro	Brazil
**3**	4	Pérez-Pérez, E.	Laboratory of Biotechnological and Molecular Analysis, Faculty of Pharmacy and Bioanalysis, Universidad de Los Andes, Mérida	Venezuela
**4**	3	Agussalim	Department of Animal Nutrition and Feed Science, Faculty of Animal Science, Universitas Gadjah Mada, Jl. Fauna No. 3, Bulaksumur, Yogyakarta	Indonesia
**5**	3	Belina-Aldemita, M.D.	Institute of Chemistry, College of Arts and Sciences, University of the Philippines Los Banos, College, Laguna	Philippines
**6**	3	D’Amico, S.	Institute for Animal Nutrition and Feed, AGES—Austrian Agency for Health and Food Safety, Spargelfeldstrase 191, Vienna, 1220	Austria
**7**	3	Pedro, S.R.M.	Biology Department, Faculty of Philosophy, Science and Letters, Universidade de São Paulo, Ribeirão Preto, SP	Brazil
**8**	3	Schreiner, M.	Department of Food Science and Technology, BOKU—University of Natural Resources and Life Sciences Vienna, Muthgasse 18, Vienna	Austria
**9**	3	Yudin, A.S.M.	Meliponini Engineering Laboratory (MePEL), Faculty of Mechanical and Automotive Engineering Technology, Universiti Malaysia Pahang, Pekan, Pahang	Malaysia
**10**	2	Basrawi, F.	Meliponini Engineering Laboratory (MePEL), Faculty of Mechanical and Automotive Engineering Technology, Universiti Malaysia Pahang, Pekan, Pahang	Malaysia

^1^ NP: number of publications.

**Table 7:** Number of documents on pot-pollen research since 2014, ranking top ten most productive institutions worldwide.

Ranking	NP ^1^	Pot-Pollen Research	
		Institution	Country
**1**	7	Universidad de Los Andes, Merida	Venezuela
**2**	7	The University of Sydney	Australia
**3**	5	Universidade de São Paulo	Brazil
**4**	4	Fundacao Oswaldo Cruz	Brazil
**5**	3	Austrian Agency for Health and Food Safety	Austria
**6**	3	Universidade Federal do Reconcavo da Bahia	Brazil
**7**	3	Universitat fur Bodenkultur Wien	Austria
**8**	3	Universitas Gadjah Mada	Indonesia
**9**	3	University of the Philippines Los Banos	Philippines
**10**	3	Universiti Malaysia Pahang Al-Sultan Abdullah	Malaysia

^1^ NP: number of publications.

**Table 8:** Top ten countries who are most productive in stingless bee pollen and pot-pollen research.

Ranking	NP ^1^	Pot-Pollen Research
		Country
**1**	15	Brazil
**2**	8	Australia
**3**	7	Venezuela
**4**	4	Indonesia
**5**	4	Malaysia
**6**	3	Austria
**7**	3	Ecuador
**8**	3	Mexico
**9**	3	Philippines
**10**	2	Argentina

^1^ NP: number of publications.

**Table 9:** Most productive sources hosting pot-pollen research since 2014.

Ranking	NP ^1^	Pot-Pollen Research
		Sources (h Index, Quartile, Impact Score) Publisher, Country ^2^
**1**	16	*Pot Pollen In Stingless Bee Melittology* Springer, Switzerland
**2**	3	*Iop Conference Series Materials Science and Engineering* (h 54, discontinued, 0.50) IOP Publishing Ltd., United Kingdom
**3**	2	*Journal of Apicultural Research* (h 66, Q2, 2.08) Taylor and Francis Ltd., United Kingdom
**4**	2	*Livestock Research for Rural Development* (h 35, Q3, 0.56) Centro para la Investigacion en Sistemas Sostenibles de Produccion Agropecuaria, Colombia
**5**	2	*Neotropical Entomology* (h 54, Q2, 1.89) Springer US, United States
**6**	1	*Biodiversitas* (h 22, Q3, 1.50) Biology department, Sebelas Maret University Surakarta, Indonesia
**7**	1	*BMC Ecology and Evolution* (h 138, Q1, 2.69) BioMed Central Ltd., United Kingdom
**8**	1	*Bodenkultur* (h 20, Q4, 0.26) De Gruyter Open Ltd., Germany
**9**	1	*Drying Technology* (h 102, Q1, 4.56) Taylor and Francis Ltd., United States
**10**	1	*Emirates Journal of Food and Agriculture* (h 37, Q3, 1.31) United Arab Emirates University, UAE

^1^ NP: number of publications; ^2^ https://www.resurchify.com.

**Table 10:** Most supportive funding sponsors of research projects on pot-pollen since 2014.

Ranking	NP ^1^	Pot-Pollen Research	
		Funding Sponsor	Country
**1**	24	Conselho Nacional de Desenvolvimento Científico e Tecnológico	Brazil
**2**	5	Coordenação de Aperfeiçoamento de Pessoal de Nível Superior	Brazil
**3**	4	Universiti Malaysia Pahang	Malaysia
**4**	3	Bundesministerium für Wissenschaft, Forschung und Wirtschaft	Austria
**5**	3	Ministry of Higher Education, Malaysia	Malaysia
**6**	3	Österreichische Agentur für Internationale Mobilität und Kooperation in Bildung, Wissenschaft und Forschung	Austria
**7**	2	Fundação de Amparo à Pesquisa do Estado do Amazonas	Brazil
**8**	2	Fundação de Amparo à Pesquisa do Estado de São Paulo	Brazil
**9**	1	Carlsbergfondet	Denmark
**10**	1	Consejo Nacional de Investigaciones Científicas y Técnicas	Argentina

^1^ NP: number of publications.

**Table 11:** Scopus subject areas of publications on pot-pollen research since 2014.

Ranking	NP ^1^	Pot-Pollen Research
		Scopus Subject Area
**1**	33	Agricultural and Biological Sciences
**2**	20	Engineering
**3**	19	Biochemistry, Genetics, and Molecular Biology
**4**	17	Environmental Science
**5**	4	Materials Science
**6**	2	Chemistry
**7**	1	Immunology and Microbiology
**8**	1	Multidisciplinary
**9**	1	Nursing
**10**	1	Pharmacology, Toxicology, and Pharmaceutics

^1^ NP: number of publications.

**Table 12:** Bibliometric descriptors of published research on direct injection food flavor from 1976 to 2023.

Bibliometric Descriptor	Counts
	All Documents
Time span	1976:2023
Scopus database	
Number of documents	48
Number of articles	38
Number of book chapters	1
Number of conference papers	2
Number of reviews	7
Number of languages	3
*Bibliometrix*	
Annual growth rate (%)	3.89
Sources (no. journals, books)	36
Author’s keywords DE (no.)	207
Keywords Plus ID (no.)	740
Average citations per document	36.73
Document average age (years)	14
Authors (no.)	190
Single-authored documents (no.)	1
Multi-authored documents (no.)	189
International co-authorship (%)	22.92
Average co-authors per document (no.)	4.81
References (total no.)	2.169

**Table 13:** Top ten most productive authors in direct injection in food flavor from 1976 to 2023 with their affiliations and countries.

Ranking	NP ^1^	Direct Injection in Food Flavor Research		
		Author	Affiliation, City	Country
**1**	8	Biasioli, F.	Department of Food Quality and Nutrition, Research and Innovation Centre, Fondazione Edmund Mach (FEM), Italy	Italy
**2**	6	Gasperi, F.	Department of Food Quality and Nutrition, Research and Innovation Centre, Fondazione Edmund Mach (FEM), via E. Mach 1, San Michele all’Adige, 38010, Italy	Italy
**3**	4	Cappellin, L.	Department of Food Quality and Nutrition, Research and Innovation Centre, Fondazione Edmund Mach (FEM), via E. Mach, 1, S. Michele, 38010, Italy	Italy
**4**	4	Capozzi, V.	Department of Food Quality and Nutrition, Research and Innovation Centre, Fondazione Edmund Mach (FEM), via E. Mach 1, San Michele all’Adige, 38010, Italy	Italy
**5**	3	Aprea, E.	Department of Food Quality and Nutrition, Research and Innovation Centre, Fondazione Edmund Mach (FEM), via E. Mach 1, San Michele all’Adige, 38010, Italy	Italy
**6**	3	Khomenko, I.	Department of Food Quality and Nutrition, Research and Innovation Centre, Fondazione Edmund Mach (FEM), via E. Mach 1, San Michele all’Adige, 38010, Italy	Italy
**7**	3	Le Quere, J.L	Centre des Sciences du Goût et de l’Alimentation (CSGA), CNRS, INRAE, Institut Agro, Université de Bourgogne, Dijon, F-21000, France	France
**8**	3	Mark, T.D.	Institut für Ionenphysik und Angewandte Physik, Leopold-Franzens Universität Innsbruck, Technikerstr. 25, Innsbruck, 6020, Austria	Austria
**9**	3	Romano, A.	Faculty of Science and Technology, Free University of Bolzano, Bolzano, 39100, Italy	Italy
**10**	3	Scampicchio, M.	Faculty of Science and Technology, Free University of Bolzano, Bolzano, 39100, Italy	Italy

^1^ NP: number of publications.

**Table 14:** Number of documents on direct injection in food flavor analysis from 1976 to 2023, ranking top ten most productive institutions worldwide.

Ranking	NP ^1^	Direct Injection in Food Flavor Research	
		Institution	Country
**1**	8	Fondazione Edmund Mach	Italy
**2**	4	Universita degli Studi di Foggia	Italy
**3**	4	Universitat Innsbruck	Austria
**4**	3	Centre des Sciences du Godt et de Alimentation	France
**5**	3	Free University of Bozen-Bolzano	Italy
**6**	3	CNRS Centre National de la Recherche Scientifique	France
**7**	2	AgroParisTech	France
**8**	2	Centre INRAE Bourgogne-Franche-Comte	France
**9**	2	Cornell University	United States
**10**	2	L’lnstitut Agro Dijon	France

^1^ NP: number of publications.

**Table 15:** Number of documents in the ten countries most productive in direct injection in food flavor research from 1976 to 2023.

Ranking	NP ^1^	Direct Injection in Food Flavor Research
		Country
**1**	10	United States
**2**	9	Italy
**3**	8	France
**4**	7	China
**5**	5	Spain
**6**	4	Austria
**7**	3	The Netherlands
**8**	2	Belgium
**9**	2	Canada
**10**	2	India

^1^ NP: number of publications.

**Table 16:** Most productive sources hostng direct injection in food flavor research from 1976 to 2023.

Ranking	NP ^1^	Direct Injection in Food Flavor Research
		Sources (h Index, Quartile, Impact Score) Publisher, Country ^2^
**1**	4	*Journal of Agricultural and Food Chemistry* (h 328, Q1, 3.21) American Chemical Society, United States
**2**	3	*Food Research International* (h 195, Q1, 8.96) Elsevier Ltd., United Kingdom
**3**	3	*Journal of Mass Spectrometry* (h123, Q3, 2.13) Wiley-Blackwell, United States
**4**	2	*Analytical and Bioanalytical Chemistry* (h 182, Q2, 4.04) Springer Verlag, Germany
**5**	2	*Food and Fermentation Industries* (h 43, Q2, 3.85) Multidisciplinary Digital Publishing Institute (MDPI), Switzerland
**6**	2	*Journal of Dairy Science* (h 216, Q1, 3.70) Elsevier Ltd., United States
**7**	2	*Molecules* (h 199, Q1, 4.71) Multidisciplinary Digital Publishing Institute (MDPI), Switzerland
**8**	2	*Trac Trends in Analytical Chemistry* (h 198, Q1, 13.53) Elsevier, the Netherlands
**9**	1	*ACS Symposium Series* (h 71, Q4, 0.66) American Chemical Society, United States
**10**	1	*American Journal of Physiology Regulatory Integrative and Comparative Physiology* (h 189, Q2, 2.58) American Physiological Society, United States

^1^ NP: number of publications; ^2^ https://www.resurchify.com.

**Table 17:** Most supportive funding sponsors of research projects on direct injection in food flavor research from 1976 to 2023.

Ranking	NP ^1^	Direct Injection in Food Flavor Research	
		Funding Sponsor	Country
**1**	2	European Regional Development Fund	European Union
**2**	2	National Institute of Food and Agriculture	United States
**3**	1	Agropolis Fondation	France
**4**	1	Chinese Academy of Sciences	China
**5**	1	Cornell University	United States
**6**	1	Department of Science and Technology, Ministry of Science and Technology, India	India
**7**	1	European Commission	European Union
**8**	1	Foundation for lchthyosis and Related Skin Types	United States
**9**	1	Horizon 2020	European Union
**10**	1	Horizon 2020 Framework Programme	European Union

^1^ NP: number of publications.

**Table 18:** Scopus subject areas of research projects on direct injection in food flavor research from 1976 to 2023.

Ranking	NP ^1^	Direct Injection in Food Flavor Research
		Scopus Subject Area
**1**	22	Agricultural and Biological Sciences
**2**	22	Chemistry
**3**	18	Biochemistry, Genetics, and Molecular Biology
**4**	5	Immunology and Microbiology
**5**	4	Chemical Engineering
**6**	4	Pharmacology, Toxicology, and Pharmaceutics
**7**	3	Neuroscience
**8**	1	Engineering
**9**	1	Environmental Science
**10**	1	Materials Science

^1^ NP: number of publications.

**Table 19:** Comparative metrics between pot-pollen (2012–2023) and direct injection food flavor publications (1976–2023).

Bibliometric Descriptor	Counts of all Documents	
	Pot-Pollen	Direct Injection Food Flavor
Time span	2014:2023	1976:2023
Scopus database		
Number of documents	40	48
Number of articles	18	38
Number of books	1	-
Number of book chapters	15	1
Number of conference papers	4	2
Number of conference reviews	1	-
Number of reviews	1	7
Subject areas		
Agricultural and Biological Sciences	33	22
Engineering	20	1
Biochemistry, Genetics, and Molecular Biology	19	18
Environmental Science	17	1
Materials Science	4	1
Chemistry	2	22
Immunology and Microbiology	1	5
Pharmacology, Toxicology, and Pharmaceutics	1	4
Chemical Engineering	-	4
Neuroscience	-	3
*Bibliometrix*		
Annual growth rate (%)	8.01	3.89
Sources (No. journals, books)	20	36
Author’s keywords DE (no.)	73	207
Keywords Plus ID (no.)	170	740
Average citations per document	6.08	36.73
Document average age (years)	3.62	14
Authors (no.)	153	190
Single-authored documents (no.)	3	1
Multi-authored documents (no.)	37	47
International co-authorship (%)	45	22.92
Average co-authors per document (no.)	4.88	4.81
References (total no.)	1976	2.169

## Data Availability

The original contributions presented in the study are included in the article, further inquiries can be directed to the corresponding author.
